# Glucose-to-Resistor Transduction Integrated into a Radio-Frequency Antenna for Chip-less and Battery-less Wireless Sensing

**DOI:** 10.1021/acssensors.2c00394

**Published:** 2022-04-07

**Authors:** Atefeh Shafaat, Rokas Žalnėravičius, Dalius Ratautas, Marius Dagys, Rolandas Meškys, Rasa Rutkienė, Juan Francisco Gonzalez-Martinez, Jessica Neilands, Sebastian Björklund, Javier Sotres, Tautgirdas Ruzgas

**Affiliations:** † Department of Biomedical Science, Faculty of Health and Society, Malmö University, Malmö 205 06, Sweden; ‡ Biofilms−Research Center for Biointerfaces, Malmö University, Malmö 205 06, Sweden; § State Research Institute, Centre for Physical Sciences and Technology, Saulėtekio av. 3, Vilnius LT-10257, Lithuania; ∥ Institute of Biochemistry, Life Sciences Centre, Vilnius University, Saulėtekio al. 7, Vilnius LT-10223, Lithuania; ⊥ Faculty of Fundamental Sciences, Vilnius Gediminas Technical University, Saulėtekio al. 11, Vilnius LT-10223, Lithuania; # Department of Oral Biology, Faculty of Odontology, Malmö University, Malmö 205 06, Sweden

**Keywords:** Internet of Things, wireless detection of glucose, direct electron transfer, glucose dehydrogenase, chip-less wireless sensing

## Abstract

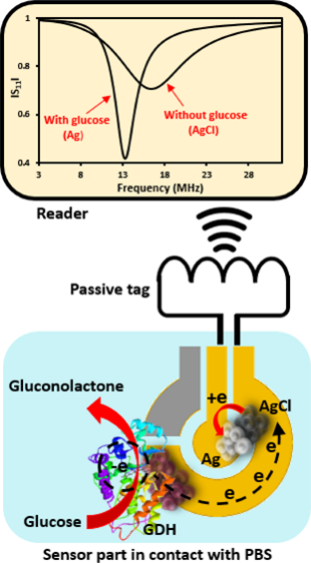

To maximize the potential of 5G infrastructure in healthcare, simple integration of biosensors with wireless tag antennas would be beneficial. This work introduces novel glucose-to-resistor transduction, which enables simple, wireless biosensor design. The biosensor was realized on a near-field communication tag antenna, where a sensing bioanode generated electrical current and electroreduced a nonconducting antenna material into an excellent conductor. For this, a part of the antenna was replaced by a Ag nanoparticle layer oxidized to high-resistance AgCl. The bioanode was based on Au nanoparticle-wired glucose dehydrogenase (GDH). The exposure of the cathode-bioanode to glucose solution resulted in GDH-catalyzed oxidation of glucose at the bioanode with a concomitant reduction of AgCl to highly conducting Ag on the cathode. The AgCl-to-Ag conversion strongly affected the impedance of the antenna circuit, allowing wireless detection of glucose. Mimicking the final application, the proposed wireless biosensor was ultimately evaluated through the measurement of glucose in whole blood, showing good agreement with the values obtained with a commercially available glucometer. This work, for the first time, demonstrates that making a part of the antenna from the AgCl layer allows achieving simple, chip-less, and battery-less wireless sensing of enzyme-catalyzed reduction reaction.

Development of wireless sensors and biosensors is intensive, and a high number of efforts are devoted to demonstrate their application as non-invasive wearables,^1,2^ implants,^3^ and highly mobile point-of-care devices.^4^ Integration of wireless biosensors into the Internet of Things (IoT), together with artificial intelligence (AI) analytics and unprecedented connectivity via the 5G infrastructure, is broadly recognized as a life-changing technical evolution.^5^ To adhere to this wireless monitoring evolution, biosensor developments exploit nanotechnologies for making smaller biosensor electrodes and miniaturized, chip-based potentiostats, which are then integrated with passive or active wireless communication techniques, such as Bluetooth, ZigBee, radio-frequency identification (RFID), and near-field communication (NFC).^6,7^ These wireless biosensor configurations require batteries and chips, which need to be reused or recycled, thus limiting a broad application of biosensors in IoT.^8^ Massive biosensor application in IoT would strongly benefit from biosensors operating in passive, chip-less, and battery-less modes. However, the availability of such wireless designs is still limited.^9^ In this work, we, for the first time, demonstrate that making a part of an antenna comprised of a AgCl layer allows achieving simple, chip-less, and battery-less wireless sensing of enzyme-catalyzed reduction reaction. We exemplify the mechanism and operation of the design by wireless detection of glucose.

The development of battery-less, or self-powered, biosensors has greatly benefitted from research on biofuel cells (BFCs).^10−12^ The concepts of BFCs can be adopted to perform biosensing and other bioelectronic functions.^13^ In this case, the bioanode or the biocathode of the BFC works as a sensing electrode, while the second BFC electrode serves as an electron collector or electron provider.^14^ Since BFCs generate low electrical power in the range of a few hundred μW, they are often combined with capacitive charging/discharging to power wireless communication.^15,16^ More simple electrical circuits have been proposed, which combine self-powered measurements and wireless signal transfer by original bioradiotransmitter design.^17^ Attempts are also made to develop chip-less biosensor tags.^18,19^ In these biosensors, specific transduction reactions modulate capacitive or resistive characteristics of the sensor material, which is intimately coupled to the tag antenna.^20−26^ To the best of our knowledge, wireless sensing based on biological AgCl reduction to Ag, as described in this work, has not been demonstrated.

Novel wireless biosensor concepts are often studied in a format of proof-of-concept glucose biosensors. Approximately one-third of academic publications in the field of wireless sensing have been focused on healthcare, where developments of wireless glucose biosensors account for a considerable share.^7^ This is fueled by the estimate that diabetes will cause a drop in GDP (gross domestic product) for OECD (Organisation for Economic Co-operation and Development) countries in order of 490 billion US$ by 2030.^27^ Thus, convenient, simple, and inexpensive wireless glucose biosensors have an undisputed and growing demand. Currently, the majority of wireless glucose biosensors are based on small wireless potentiostats,^28−33^ including the well-established and clinically viable continuous glucose monitoring systems.^34^ Although these biosensors can be made impressively small,^30^ they contain a number of semiconductor elements, which are generally expensive and associated with low disposability and sustainability. Simpler passive, battery-less, and chip-less designs have been realized on magnetoelastic pH-sensitive material^35^ and phenol-boronic acid hydrogel.^36^ The possibility of optical (wireless) monitoring of glucose-based oxygen-sensitive polymer implanted under the skin was also demonstrated.^37^ These examples comprise too small number of ideas for building a creative research arena, which could enable development of simpler wireless glucose biosensors. To augment this research field, we disclose simple and novel wireless glucose biosensor design.

In our recent work, we have demonstrated that direct electronic coupling of the enzymes to a nanomaterial, constituting part of the tag antenna circuit, can be a basis for the construction of wireless biosensors.^23^ The proof of concept has been illustrated for wireless detection of hydrogen peroxide (H_2_O_2_) by utilizing silver nanoparticles (AgNPs) as part of the tag antenna, which were oxidized to AgCl by H_2_O_2_, catalyzed by horseradish peroxidase.^25^ Although the response time of the biosensor was in the range of hours, this work disclosed wireless monitoring of Ag/AgCl redox conversion, driven by enzymatic redox reactions, as one of the simplest concepts of wireless biosensing to date. However, this simple design concept has not been demonstrated for monitoring of AgCl reducing reactions, e.g., detection of glucose.

The aim of this work was to demonstrate that wireless glucose detection can be realized by exploiting direct electron coupling of glucose dehydrogenase (GDH) to the nanomaterial integrated into a tag antenna circuit, i.e., a radio-frequency (RF) antenna. To realize this, the AgCl layer was included as part of the antenna of the biosensor tag. In the presence of glucose, the enzyme catalyzed oxidation of glucose and provided electrons for AgCl reduction to Ag. The Ag/AgCl redox conversion was wirelessly monitored, thus enabling wireless detection of glucose. The obtained results, in the frame of our earlier publications, prove that the proposed design of the wireless biosensor, where enzymes are electronically coupled to materials of the antenna, is generic and possible to realize with different oxidoreductase enzymes. Here, for the first time, we present a comprehensive description of this type of wireless biosensor in terms of its construction, the principle of operation, and electrical equivalent circuit. Glucose-to-resistor transduction, which exploits Ag/AgCl redox reaction on the part of the tag antenna and enables wireless glucose detection, is also presented for the first time. All this is important for future improvement and optimization of this wireless biosensor concept. To mimic the final application, the proposed biosensor was evaluated through wireless measurements of glucose in whole blood. In the developed form, the bioanode-cathode-antenna part of the biosensor could be considered as having a shape/form similar to a glucose strip. This antenna-containing strip, allowing wireless detection, could also bring an advantage by excluding the need to connect the strip to the measuring device. Such a biosensor option might provide extra convenience to the users who experience difficulties to insert a glucose biosensor strip into the potentiostat. In general, the results of this work suggest that the realization of intimate enzyme-antenna connection, and thus, development of simple wireless biosensors, can possibly be achieved with a number of other enzymes since direct electron transfer reactions have been shown for a high number of oxidoreductases.^38^

## Results and Discussion

**Scheme 1:**
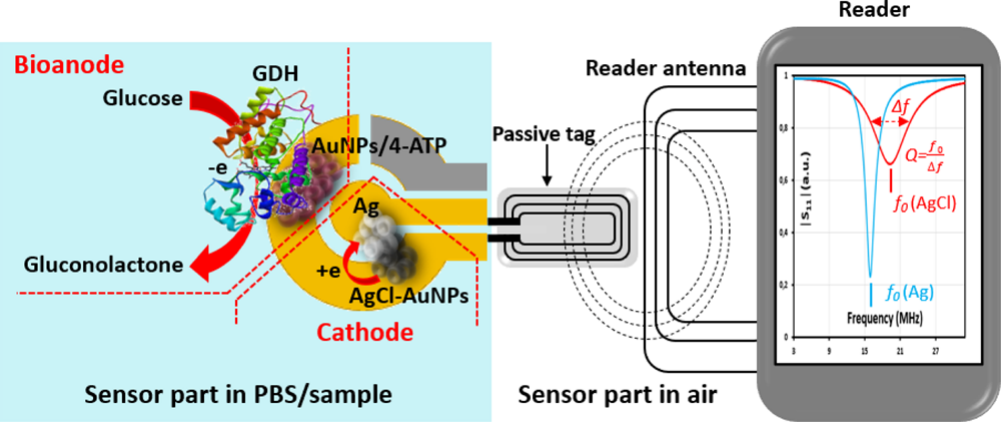
Conceptual Illustration of a Battery-less and Chip-less Biosensor Tag for Wireless Measurement of Glucose in Solution The design is comprised of (i) a bioanode, (ii) a cathode layer, and (iii) a passive tag antenna, with a 5 mm removed antenna line, allowing connection of two electrodes of SPE, bridged by the cathodic transduction layer. The bioanode is based on glucose dehydrogenase in direct electron transfer contact with one of SPE electrodes. The cathode layer contains AgNPs, which were electrooxidized to AgCl before measurements of glucose. The SPE-antenna coupling makes the cathode layer a part of the tag antenna circuit. The tag reader wirelessly reads the impedance characteristics of the antenna circuit, which are represented by the reflection spectrum, |*S*_11_|. This |*S*_11_| provides easy reading of the characteristic frequency, *f*_0_, and the corresponding *Q*-factor of the antenna circuit. Determining the change of the *f*_0_ and *Q*-factor makes it possible to monitor the resistance change of the transduction layer when the AgCl layer transforms to metallic Ag. This transformation happens when the SPE is exposed to glucose solution, since oxidation of glucose on the bioanode reduces AgCl to Ag on the cathode. The SPE-tag antenna connection thus allows wireless detection of glucose.

Scheme 1 illustrates the working principle of the developed biosensor for wireless detection of glucose by showing coupling of a cathodic AgCl layer into the antenna of the RF tag and a sensing bioanode, which, in the presence of glucose, reduces AgCl (cathode material) to Ag.

An important feature of the biosensor tag is that the cathode layer bridges two electrodes of the SPE (working and counter electrodes in this particular design) and that the connection of these electrodes to the tag makes the cathode layer a part of the tag antenna circuit. The mechanism of wireless glucose detection relies on the bioelectrocatalytic oxidation of glucose at the bioanode with a consequent supply of electrons to the cathode. This reduces AgCl in the cathode layer to metallic Ag. As a result, the transition of a poor electric conductor (AgCl layer) to an excellent conductor (metallic Ag) is accomplished. This transition strongly lowers the resistance (generally impedance) of the tag antenna circuit. Importantly, these reactions and the bioanode-cathode connection thus realize glucose-to-resistance transduction. The impedance change is then wirelessly detected by measuring the characteristic frequency, *f*_0_, and *Q*-factor of the biosensor-tag system. Practically, *f*_0_ and *Q*-factor are determined from the |*S*_11_| spectrum, which is measured by using a wireless tag reader (the |*S*_11_| measurements are explained in Experimental Section). In the following paragraphs, the essential components and features of this wireless biosensor design are described. This includes a description of the bioanode and cathode, proof-of-concept demonstration of glucose detection and a calibration plot, the effect of chloride concentration on the response, and presentation of the equivalent circuit of the biosensor tag. The majority of experiments have been made to explain the concept of this novel biosensor design and to demonstrate possible means to optimize and improve the performance characteristics of this battery-less, chip-less wireless biosensor design.

### GDH in Direct Electron Transfer (DET) on the Electrode Surface Constitutes a Bioanode

**Figure 1:**
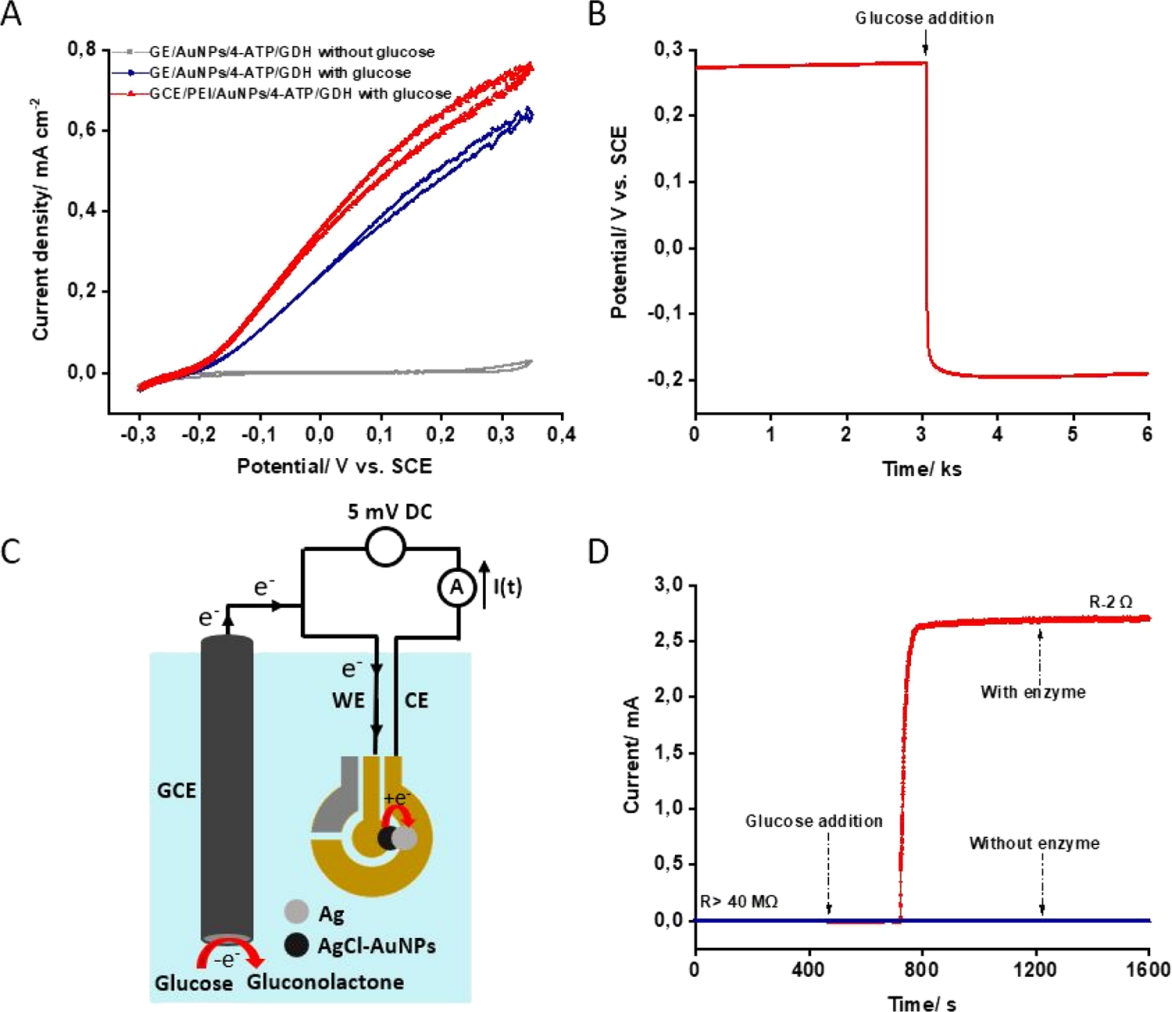
(A) Cyclic voltammograms of GE/AuNP/4-ATP/GDH- and GCE/PEI/AuNP/4-ATP/GDH-modified bioanode electrodes recorded in PBS, pH 7.4, in the absence and presence of glucose (50 mM). Potential scan rate of 1 mV s^–1^ starting at −0.3 V. (B) Open-circuit potential (OCP) recorded for the AuNP/4-ATP/GDH electrode before and after addition of 50 mM glucose. (C) Schematic presentation of connection of the bioanode (GDH-modified) and the cathode (SPE hosting the AgCl-containing transduction layer). (D) Amperometric curves (current vs time) obtained with the electrodes, connected as shown in panel (C) and immersed in PBS. Glucose addition gave a 50 mM glucose concentration in PBS. This allowed the electrons, generated at the bioanode, to convert AgCl to Ag on the cathode layer. The AgCl/Ag conversion is obvious from the increase in current through the cathodic transduction layer (red trace). The control experiment shows that the bioanode without GDH was not able to convert AgCl to Ag; the current through the transduction layer is the same after the addition of glucose (blue trace).

To realize wireless glucose biosensing, GDH was immobilized on a gold nanoparticle (AuNP)-modified planar gold electrode (GE) or a glassy carbon electrode (GCE) following a previously described procedure.^39^ AuNPs were utilized to increase the surface area, while 4-ATP (4-aminothiophenol) was used to immobilize GDH in DET-facile orientation (these modifications are detailed in the Supporting Information). The purpose of using the GCE was to provide a higher geometric area, as compared to the area of the planar GE. The resulting glucose-sensing electrodes are denoted as GE/AuNP/4-ATP/GDH and GCE/PEI/AuNP/4-ATP/GDH. Cyclic voltammetry measurements (Figure 1A) proved that, in the absence of glucose, the electrodes showed no catalytic current. In the presence of glucose (50 mM), the bioelectrocatalytic oxidation of glucose started at −0.23 V (vs SCE), proving a successful DET coupling of GDH to the electrode surface. Maximal bioelectrocatalytic current densities, at 50 mM glucose, were 0.63 and 0.75 mA cm^–2^ for GE/AuNP/4-ATP/GDH and GCE/PEI/AuNP/4-ATP/GDH, respectively. The open-circuit potential (OCP vs SCE) of the bioanode in the presence of glucose corresponded to cyclic voltammetry results (Figure 1A) and was equal to −0.2 V (Figure 1B). This relatively high negative OCP is an important feature for the electrode to function as a bioanode in the novel glucose-to-resistor transduction. It should be emphasized that, without 4-ATP modification of AuNPs, the bioelectrocatalytic current was 15 times lower, specifically, 0.05 mA cm^–2^ (compared to 0.75 mA cm^–2^; see the Supporting Information). These results confirmed the essential role of 4-ATP for the enzyme immobilization in DET-facile orientation.^39−41^ Importantly, the GE/AuNP/4-ATP/GDH and GCE/PEI/AuNP/4-ATP/GDH electrodes gave similar current densities, indicating the robustness of the electrode preparation procedure. Additionally, both electrode designs showed excellent stability and allowed their use as glucose-sensing bioanodes for more than a week without losing bioelectrocatalytic activity (kept in phosphate-buffered saline (PBS), at +4 °C when not in use). To emphasize the importance of AuNP/4-ATP modification in achieving DET of GDH, these GDH-modified electrodes, in the text below, are referred to as AuNP/4-ATP/GDH-modified electrodes.

### AgCl Layer Is a Part of the Tag Antenna Circuit and Functions as a Cathode in Glucose-to-Resistance Transduction

To demonstrate that the AgCl-containing layer between two electrodes on the SPE (see Scheme 1) acts as a cathode, a complete design of the wireless biosensor (Scheme 1) is inconvenient. Advantageously, the construction can be divided into separate components for easier study of their functionality. Figure 1C depicts the bioanode and cathode separated from the RF antenna and connected to a potentiostat. Specifically, Figure 1C depicts a cathode, comprised of a AgCl layer, which bridges two electrodes on the SPE in connection with the GDH-based bioanode. The bridge accomplishes glucose-to-resistance transduction; if the layer is dominated by AgCl, the resistance is high. However, if the bioanode reduces AgCl to metallic Ag, the resistance of the layer becomes very low. The resistance change of the transduction layer was measured by a potentiostat, in amperometric mode, as shown in Figure 1C.

In Figure 1C, the AuNP/4-ATP/GDH electrode is connected as a bioanode in respect to a cathode comprised of a AgCl-containing transduction layer. This connection enables glucose-powered reduction of AgCl to metallic Ag. For practical demonstration, the electrodes were immersed in PBS, 5 mV DC potential was applied on the AgCl-containing transduction layer, and the resulting current was registered (see data in Figure 1D). The figure shows that the current is very different before and after the addition of 50 mM glucose in PBS. It can also be noted that, in this particular example, it took 264 s until the low initial current (0.125 nA, resistance of the transduction layer *R* = 40 MΩ) changed to 2.68 mA, indicating that AgCl on the cathode has been reduced to metallic Ag by glucose oxidation at the bioanode. The layer resistance became equal to 2 Ω as calculated from the amperometric data shown in Figure 1D. This experiment confirms that the GDH-modified electrode acts as a bioanode and the AgCl-containing layer as a cathode. Importantly, the experiments prove that the described connection and modification of the electrodes provide glucose-to-resistance transduction.

### Proof-of-Concept Demonstration of Wireless Detection of Glucose

**Figure 2:**
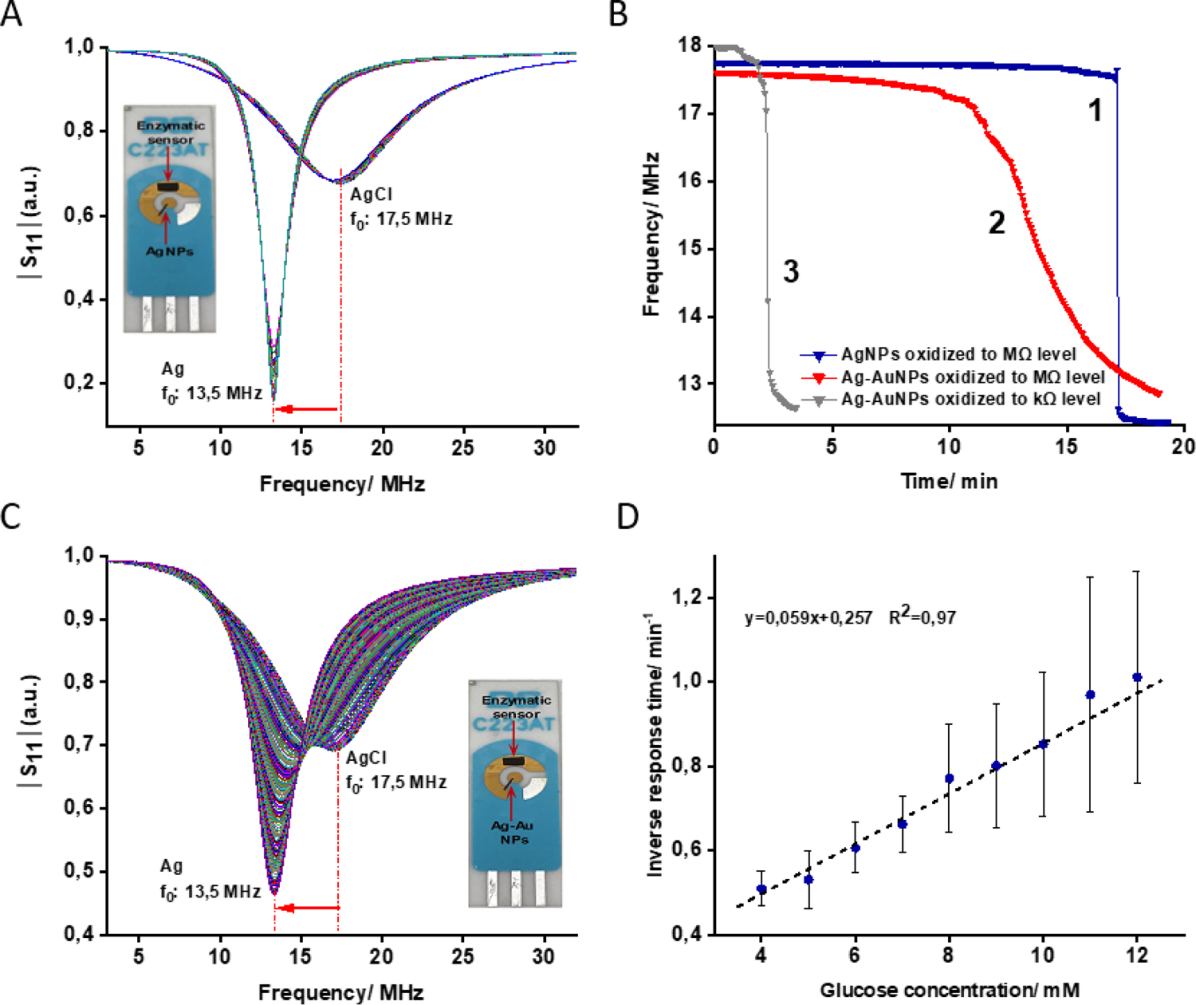
(A) Dependence of the reflection coefficient |*S*_11_| on frequency recorded with wireless biosensor configuration shown in Scheme 1. The curves are recorded for the biosensor setup where the transduction layer on the SPE was comprised of only AgNPs converted to AgCl (marked with AgCl) and after the AgCl reduction to metallic Ag (marked with Ag) by the bioanode. (B) Change of characteristic frequency of the wireless biosensor as a response to 4 mM glucose (time zero represent time when glucose was added into the measurement cell). Notes indicate the composition of the transduction layers and their resistances after the AgNP oxidation to AgCl. (C) |*S*_11_| vs frequency recorded for the biosensor where the transduction layer was comprised (marked with AgCl) of AuNP-AgNP mixture with the AgNPs converted to AgCl and (marked with Ag) after the AgCl reduction to metallic Ag. The reduction reactions were driven by the bioanode in the presence of 4 mM glucose in PBS. Different |*S*_11_| curves reflect monotonic |*S*_11_| transition in time. The corresponding *f*_0_ vs *t* trace is shown in panel (B), curve 2. (D) Calibration curve, i.e., the inverse response time of the biosensor tag vs glucose concentration in PBS. The transduction layer was comprised of AgNP-AuNP mixture where AgNPs were electrochemically oxidized to AgCl. The wireless biosensor configuration is depicted in Scheme 1, where only the SPE with the bioanode and cathode (the transduction layer) was in solution during the measurements of glucose.

To demonstrate wireless glucose sensing, AuNP/4-ATP/GDH bioanode design was realized on the counter electrode of the SPE as shown in Figure 2A (inset; left). For the initial proof-of-concept experiments, the working and counter electrodes on the SPE were bridged by drop-casting a layer comprised solely of AgNPs (0.5 μL, three times deposition; insets in Figure 2A). The AgNP layer was electrochemically oxidized in PBS to AgCl and then the SPE was connected with the RF antenna as shown in Scheme 1. The SPE was immersed in PBS and the |*S*_11_| characteristic curve (marked with AgCl in Figure 2A) of this biosensor was wirelessly recorded using the RF antenna reader. The |*S*_11_| indicated that the SPE-antenna-coupled circuit had a characteristic frequency, *f*_0_, equal to 17.5 MHz. After the addition of glucose into the PBS solution, the |*S*_11_| transformed (after some time) into a new |*S*_11_| trace, marked with Ag in Figure 2A. This change of |*S*_11_| indicates that the electrical antenna circuit has changed, causing a new value of a characteristic frequency of 13.5 MHz. As discussed above, this is a consequence of the change of the resistance of the transduction layer after AgCl is reduced to Ag by glucose oxidation on the bioanode. This result provides a proof of concept of wireless detection of glucose and confirms the role of the transduction layer as an enabler of wireless registration of enzymatic redox reaction. It could be noted that the value of the characteristic frequency (13.5 MHz) is very close to the value of the characteristic frequency of the original RF antenna of this particular NFC tag (13.56 MHz tag). This is only possible if the transduction layer after AgCl reduction to Ag has negligible resistance compared to the resistance of the tag antenna; the relation of the transduction layer resistance to |*S*_11_| characteristics (*f*_0_ and *Q*-factor) is discussed further.

### Optimization of the Wireless Biosensor Response

The change of *f*_0_ as a response to glucose, demonstrated in Figure 2A, provides evidence that the biosensor system allows wireless detection of glucose in solution. It is also clear that the higher is the glucose concentration, the shorter is the response time. However, a more extensive study showed that the response time also depended on the amount of AgNPs deposited in the transduction layer and the fraction of their conversion to AgCl (e.g., complete or partial AgNP layer oxidation; see Experimental Section). We also noticed that the microstructure and composition of the transduction layer (e.g., solely AgCl or AuNP-AgCl mixture) affected the response time. We studied the microstructure by impedance spectroscopy; however, it was not possible to simply relate the results to the response of the wireless biosensor (impedance characterization of the microstructure of the layers is presented in the Supporting Information). Some of the features of the transduction layer, which enable tuning of the wireless biosensor characteristics, such as response time and sensitivity, are discussed below.

To understand how the response time of the proposed wireless biosensor can be tuned by manipulating the AgCl-containing transduction layer, two aspects have been assessed. Particularly, (i) the effect of AgNP oxidation to AgCl and (ii) the effect of admixing 20% of AuNPs in the transduction layer were studied. Only the most important observations are summarized below. The thicknesses of AgNP or AgNP-AuNP transduction layers drop-casted for bridging the two electrodes on the SPE were a few micrometers (see the Supporting Information). Complete or partial AgNP oxidation to AgCl in the transduction layer was achieved at 200 mV applied voltage for 120 s or 70 mV for 12 s, respectively. The layers after complete conversion of AgNPs to AgCl had a few MΩ resistance values. Partial oxidation gave the layer with a few kΩ resistance values. Figure 2B demonstrates a few examples of how the characteristic frequency of the biosensor-tag system changed in time after the SPE, hosting the bioanode and cathode with a differently oxidized transduction layer, was exposed to 4 mM glucose in PBS. As can be seen in Figure 2B, the characteristic frequency vs time curves are very different. The response time (the time needed to reach *f*_0_ close to 13.5 MHz from the initial *f*_0_ of 17.5–18 MHz) as well as the rate of the *f*_0_ change depended on the oxidation degree of the AgNPs in the layer and its composition. Specifically, for the layer comprised of just AgNPs after their complete oxidation (resistance, >40 MΩ), the response time to 4 mM glucose is equal to approximately 17.5 min (Figure 2B, curve 1). For the layer comprised of AgNP-AuNP mixture and partial AgNP oxidation (layer resistance, 2 kΩ), the response time is shorter than 3 min. For the same layer, which was completely oxidized, (*R* = 11 MΩ), the response time was prolonged to longer than 15 min. It is also obvious that, for the layers comprised of AgNP-AuNP mixture, the change of the characteristic frequency is gradual (Figure 2B, curve 2). Figure 2C shows corresponding |*S*_11_| trace transformation in due time of the experiment. The gradual change of the characteristic frequency might be beneficial in the future optimization of transduction layers since this process might indicate that Ag/AgCl reaction proceeds more homogeneously in the entire layer. In conclusion, it is important to state that partial oxidation and addition of AuNPs to the transduction layer allow a shorter response time of the wireless biosensor to glucose. The fact that the response times can be shortened by manipulating the transduction layer composition and, obviously, the amount of AgNPs in the transduction layer points to the possibility of optimizing this biosensor design for achieving more rapid wireless measurements of glucose.

### Sensitivity of the Wireless Biosensor Tag to Glucose: Calibration Curve

Data in Figure 2B demonstrate that the response time of the biosensor tag to glucose depends on several characteristics of the transduction layer. However, if the transduction layer is made by using the same method, the response time of the biosensor tag is reproducible (see the Supporting Information, Figure S9) and dependent on glucose concentration (Figure 2D). Figure 2D shows that the higher is the glucose concentration, the shorter is the response time. This dependence, inverse response time vs glucose concentration (Figure 2D), represents a calibration curve; the curve was obtained with a transduction layer comprised of 20% AuNP and 80% AgNPs (3× 0.5 μL of concentrated AuNP-AgNP mixture). The AgNPs in the layer were oxidized by applying 70 mV vs SCE for 12 s, resulting in the layer resistance of approximately 2 kΩ.

As can be seen in Figure 2D, the calibration is linear. The linearity is expected. Usually, amperometric biosensors rely on the linear dependence of current response (charge/time: *Q*/*t*) vs concentration. In our case, the charge is always the same and it is determined by the amount of AgCl in the transduction layer. So, if the current generated by the bioanode increases with glucose concentration, then a shorter time period (shorter response time) is needed to get the same amount of the charge (electrons), which reduces AgCl to Ag. This indicates that inverse response time vs concentration should be linear as has been found experimentally and shown in Figure 2D. It is important to note that, during the measurement of glucose, a high-resistance AgCl cathodic transduction layer is converted to a low-resistance Ag layer, i.e., the bioanode-cathode connection realizes glucose-to-resistance transduction. However, independent of the glucose concentration, the final resistance of the resulting Ag layer is practically the same and the time to reduce AgCl in the cathodic layer to Ag is different. To develop a practically competitive wireless biosensor, our work is directed to increase the reproducibility of AgNP deposition and to produce transduction layers containing smaller amounts of AgNPs, aiming for response times shorter than 1 min.

### The Response of the Biosensor Does Not Depend on the Chloride Concentration

**Figure 3:**
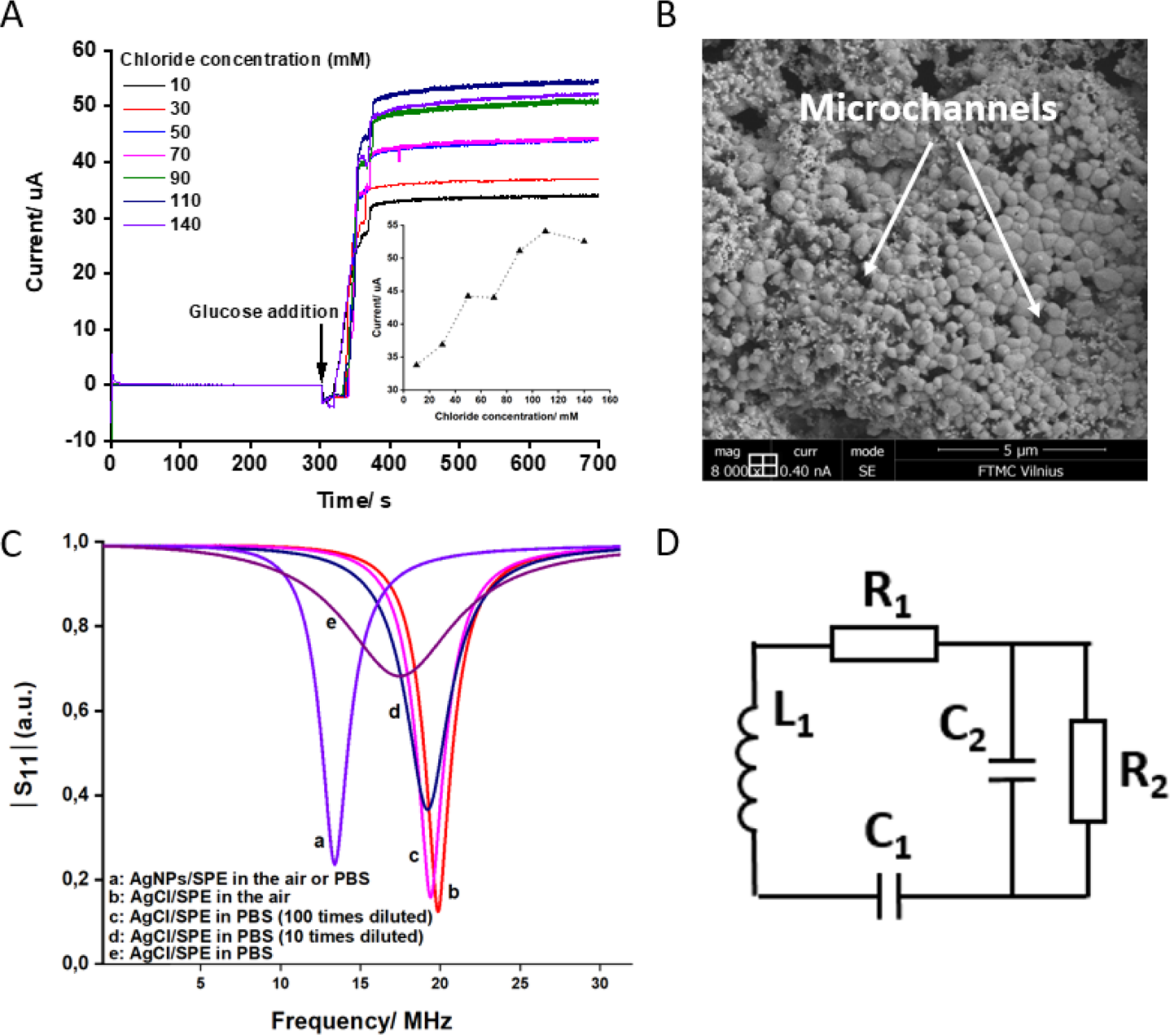
(A) Responses of the biosensor to 6 mM glucose in phosphate buffer containing different concentrations of KCl. The response was recorded using amperometric measurement mode with the electrode connection shown in Figure 1C. The transduction layer was made by drop-casting AgNP-AuNP mixture and electrooxidation of AgNPs to AgCl in PBS. Inset: dependence of maximal current that flows through the transduction layer on the chloride concentration present in the solution during the AgCl reduction to Ag by the bioanode. (B) SEM image of the transduction layer comprised of AgNP-AuNP mixture after AgNP oxidation to AgCl. (C) |*S*_11_| curves recorded with the tag-SPE system, where the transduction layer was based on AgNPs. The oxidation state of Ag in the layer and the solution in the measurement cell are specified. (D) Equivalent circuit of the biosensor tag. L_1_, R_1_, and C_1_ represent the inductance, resistance, and capacitance of the original tag antenna circuit, respectively. R_2_ and C_2_ represent the resistance of the Ag/AgCl transduction layer (including adjacent PBS solution) and the parasitic capacitance of the antenna-SPE connections, respectively.

After discussing the proof of concept (Figure 1) and the calibration curve (Figure 2D), it is important to understand if changes of solution composition (e.g., chloride concentration) might affect the biosensor response. The redox potential of Ag/AgCl increases with the decrease in chloride concentration, and thus, the thermodynamic potential difference between the bioanode (glucose/gluconolactone) and the cathode (Ag/AgCl) increases. The question is, thus, if this will affect the biosensor response time. To assess this, the response of the biosensor to the same glucose concentration (6 mM) at different concentrations of chloride present in the solution has been studied using the experimental setup shown in Figure 1C. As can be seen in Figure 3A, the amperometric responses are very similar in terms of the response time, which is equal to 43.3 ± 4.7 s, determined at 50% of the maximal current. The observed independence of the biosensor response time on the chloride concentration proves that the potential difference between the bioanode and the cathode is sufficiently high; the bioanode and cathode reactions can no longer be made more rapidly by increasing the potential difference between these electrodes, e.g., by lower chloride concentrations.

Figure 3A (inset), however, shows that the current that passes the metallic Ag transduction layer, after the biosensor responded to glucose (i.e., after the reduction of AgCl to Ag), is higher if the AgCl reduction to Ag proceeded at higher KCl concentrations. The observed dependence is most probably determined by the difference in the layer microstructures, including the possible presence of AgCl left without being reduced. The fact that the Ag layer is comprised of microstructures is obvious from SEM images presented in Figure 3B; however, the conductivity–microstructure relation is difficult to prove. Characterization of these layers using electrochemical impedance spectroscopy also confirmed that their electrochemical behavior can be described by considering the microstructure-containing microchannel (see the Supporting Information).

### AgCl-to-Ag Transition in the Transduction Layer Decouples the Wireless Biosensor from the Biosensor Environment

The cathodic transduction layer possesses an additional, very interesting, and practically important feature of the biosensor. After the biosensor response to glucose, i.e., after AgCl reduction to Ag, wirelessly monitored |*S*_11_| becomes completely insensitive to the surrounding of the SPE electrode containing the transduction layer. As shown in Figure 3C, curve a, the |*S*_11_| trace is the same if the SPE, with the transduction layer comprised of Ag, is in PBS or in air. This means that the transduction layer comprised of Ag completely decouples surrounding conditions at which the SPE with the bioanode and cathode is exposed to. The |*S*_11_| no longer depends on, e.g., the salt concentration of the solution. It could be said that the environmental and surrounding conditions are short-circuited by the metallic Ag transduction layer. Intuitively, this feature might be very important in practical applications of the biosensor. For a deeper understanding of this chip-less and wireless biosensor, tag-SPE features were modeled by an equivalent circuit shown in Figure 3D. Relevant experiments and the modeling of the equivalent circuit are described in the next paragraph.

### The Equivalent Circuit of the Wireless Biosensor Is a Tag Antenna, which Hosts Redox Reaction-to-Resistance Transduction

**Figure 4:**
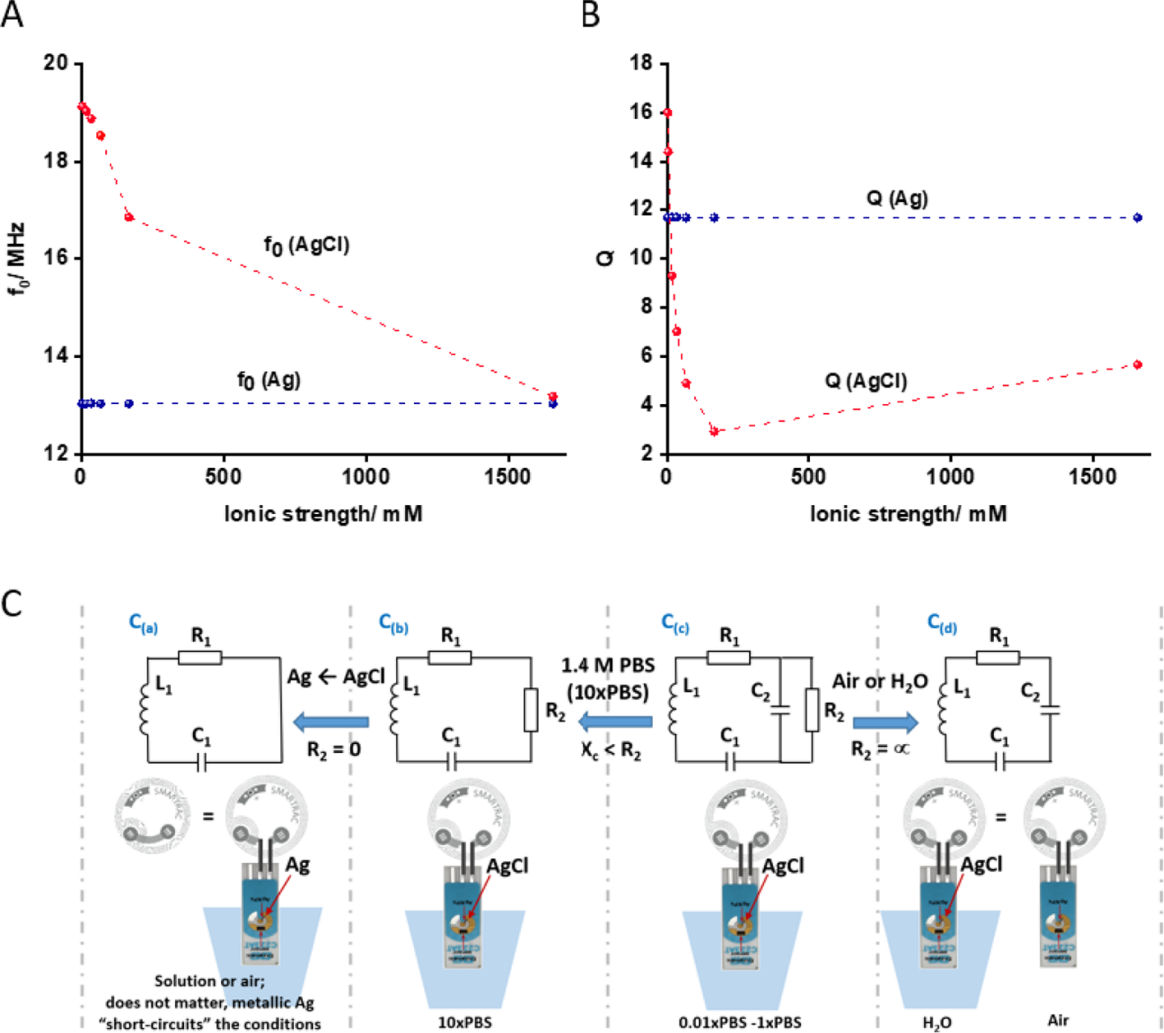
(A) Characteristic frequency (*f*_0(Ag)_ and *f*_0(AgCl)_) and (B) *Q*-factor (*Q*_(Ag)_ and *Q*_(AgCl)_) of the antenna with a coupled SPE exposed to air or immersed into solution of different ionic strengths. The SPE hosts a transduction layer comprised of Ag or AgCl. (C) Equivalent circuits representing the antenna circuit and its illustration (tag antenna and the SPE) when the transduction layer on the SPE is exposed to different environmental conditions: air, water, and concentrated or diluted PBS as indicated in the drawings.

In Figure 3D, we propose an equivalent circuit of the wireless biosensor comprised of an RF antenna of NFC tag with an integrated Ag/AgCl-based transduction layer. Since the equivalent circuit of this type of wireless biosensor has never been discussed before, an easy-to-grasp explanation is provided below. Rigorous modeling was also conducted, and it is described in the Supporting Information. To understand how the equivalent circuit in Figure 3D describes the dependence of the characteristic frequency *f*_0_ and *Q*-factor of the biosensor tag (i) on the state of the transduction layer (metallic Ag vs AgCl) and (ii) on the salt concentration of solution where the SPE with the transduction layer is immersed to, additional experiments have been done. The antenna-coupled SPE, containing a metallic Ag or AgCl transduction layer, was exposed to air or immersed into PBS of different dilutions/concentrations, and |*S*_11_| curves were recorded. An example of the |*S*_11_| curves is presented in Figure 3C; |*S*_11_| depends on the state of the transduction layer (AgCl or Ag) and on the dilution of PBS when the transduction layer is comprised of AgCl. The ionic strength dependencies of the corresponding *f*_0_ and *Q*-factor, derived from these |*S*_11_| curves, are presented in Figure 4A,B. As can be seen from these figures, the values of *f*_0_ and *Q* are completely independent of the ionic strength of the solution when the transduction layer is comprised of Ag (curves marked with *f*_0(Ag)_ and *Q*_(Ag)_, Figure 4A,B, respectively). The absence of the dependence confirms that the |*S*_11_| characteristic does not depend on the solution composition to which the metallic Ag transduction layer is exposed to. Additionally, since the *f*_0(Ag)_ and *Q*_(Ag)_ are very close to the original tag characteristics, the equivalent circuit, to the first approximation, can be represented by the usual RF tag antenna circuit, which is composed of a serial connection of tag antenna inductance (L_1_), resistance (R_1_), and capacitance (C_1_).^42−44^ The corresponding circuit is shown on the far left in Figure 4C, noted as C_(a)_.

It can be easily noted (Figure 4A,B) that, when the transduction layer is composed of AgCl and exposed to 10 times concentrated PBS (ionic strength is equal to 1657 mM), the characteristic frequency *f*_0(AgCl)_ is almost the same as for the transduction layer comprised of metallic silver, *f*_0(Ag)_ (*f*_0(AgCl)_ = 13.18 MHz and *f*_0(Ag)_ = 13.03 MHz). The *Q* value is, however, significantly lower: *Q*_(AgCl)_ = 5.66 and *Q*_(Ag)_ = 11.69. To account for the similarity of *f*_0_ and for the difference of *Q* values, the antenna-SPE circuit can be complemented by a resistor R_2_, as shown by the circuit C_(b)_ in Figure 4C. The close similarity of *f*_0_ (*f*_0(Ag)_ ≈ *f*_0(AgCl) in 10×PBS_) means that reactive elements C_1_ and L_1_ are practically the same in both these situations (see the equation for *f*_0_ in the Supporting Information, Table S4). Additionally, the R_2_ was found to be dependent only on the ionic strength of the solution since removing the AgCl transduction layer (leaving the gap between the two electrodes on the SPE empty) did not change the |*S*_11_| dependence on the ionic strength (data are not shown).

By examining the |*S*_11_| characteristics, recorded with the transduction layer comprised of AgCl, it could be noted that the *f*_0(AgCl)_ and *Q*_(AgCl)_ values are very similar in air (*f*_0(AgCl)_ = 19.67 MHz and *Q*_(AgCl)_ = 18.23) and in pure water (*f*_0(AgCl)_ = 19.12 MHz and *Q*_(AgCl)_ = 17.98; also listed in Tables S3 and S4). Under these conditions, R_2_ can obviously be regarded as infinity, which is shown in the circuit noted by C_(d)_ (far right in Figure 4C). Additionally, since the values of *f*_0(AgCl)_ in air and in water are very similar, when the relative permittivity values of these media are different (relative permittivity of air is ε_r_ = 1 and that of water is ε_r_ = 80), we can conclude that the C_2_ is very little affected by the double-layer capacitance of the transduction layer (AgCl) and the underlying surface of gold electrodes of the SPE. The value of C_2_ must thus be dominated by a parasitic capacitance of the connections and wire lines connecting the SPE to the gap of the RF antenna. The fact that the C_2_ does not depend on the double layers of the electrodes of the SPE and on the bridging AgCl layer is quite unexpected and might not be a general phenomenon (to conclude, a deeper study of the antenna-SPE system is needed).

In summary, this simplified explanation of the equivalent circuit of the wireless biosensor confirms that the Ag/AgCl transduction layer can be represented as a resistor. It is important to note that the layer can carry out transduction of any reaction that is able to achieve Ag/AgCl redox transformations. This means that the proposed Ag/AgCl layer inserted as part of the RF antenna can be regarded as a general redox reaction-to-resistance transducer. The circuit analysis (Figure 4C) is useful for explaining the specific and general features of the mechanism of the wireless biosensor. Additionally, rigorous mathematical modeling has been performed based on analytical equations, which describe the *f*_0_ and *Q* values (see the Supporting Information). The equations are complicated; however, they can be written in symbolic form (we exploited Mathematica software) and used to fit the experimental data of Figure 4A,B. Fitting is shown in Figure S10A,B, and the corresponding values of circuit elements R_1_, L_1_, C_1_, and C_2_ that had been found from this fitting were equal to 0.247 Ω, 8.23 × 10^–8^ H, 9.63 × 10^–9^ F, and 29.98 × 10^–9^ F, respectively. In general, the values look reasonable, e.g., the resistance of the tag antenna is, as expected, below 1 Ω. However, deeper analysis of the values and improvement of the equivalent circuit are needed to explain the physical origin of the circuit elements.

### Application of the Proposed Wireless Biosensor for Detection of Glucose in Whole Blood

**Figure 5:**
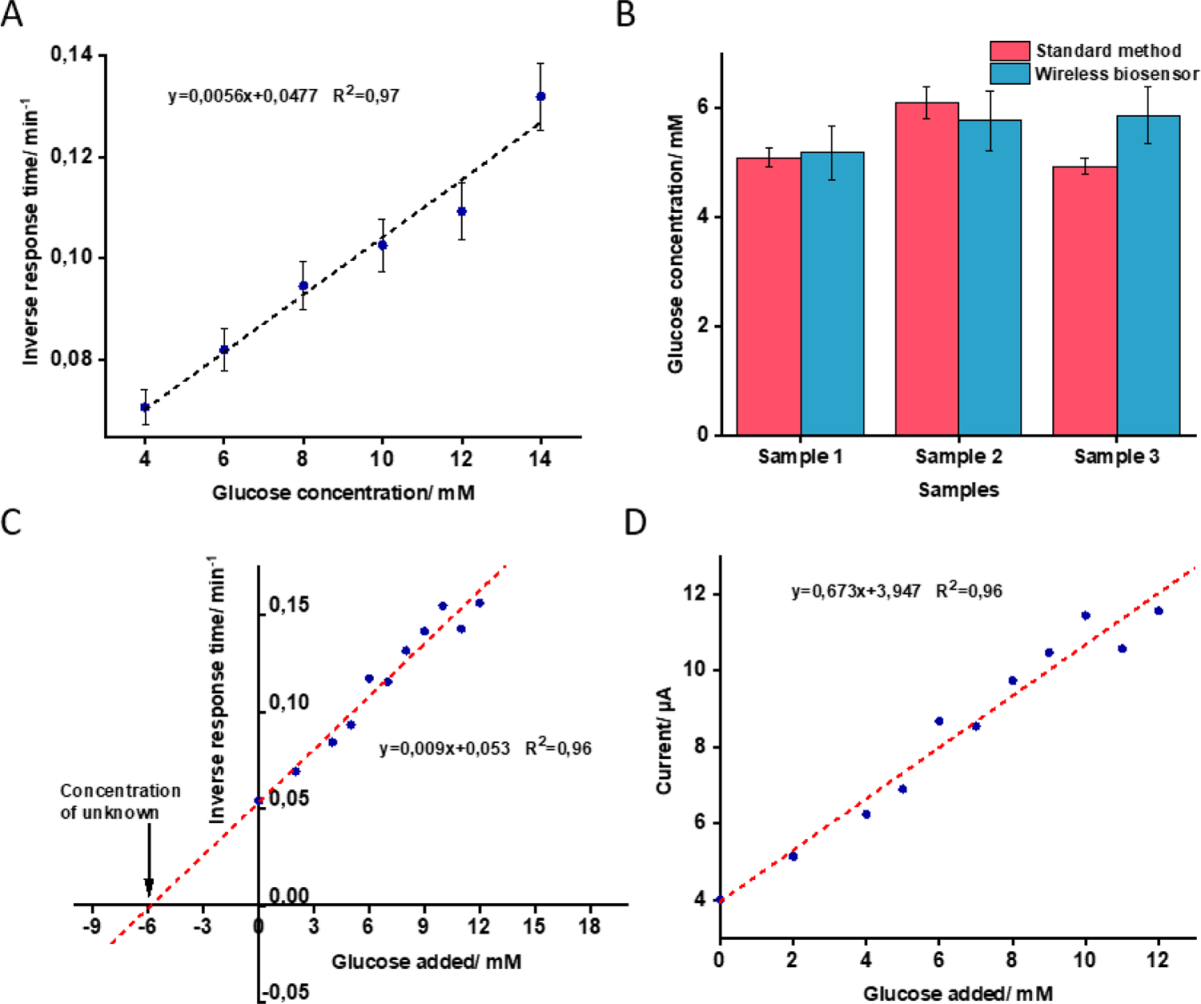
(A) Calibration curve, i.e., the inverse response time of the biosensor tag vs glucose concentration in PBS. The transduction layer was comprised of Ag-AuNP mixture electrochemically oxidized to the MΩ level. (B) Result of comparison between the proposed wireless biosensor and the standard method (glucometer) for glucose analysis in the whole blood samples from three different donors. (C) Calibration curve of the wireless biosensor obtained by the standard addition method using whole blood samples. (D) Estimation of the current generated by the enzyme biosensor during successive addition of glucose to the whole blood samples.

The application of the proposed wireless biosensor in whole blood analysis was shown by the calibration curve method and standard addition method. In the calibration curve method, the SPE modified with a bioanode and a cathode layer (Ag-AuNPs oxidized to the MΩ level) was prepared and coupled to the tag antenna and the characteristic frequency was monitored wirelessly with the SPE in PBS containing different concentrations of glucose (4–14 mM). The time taken for the transition of the characteristic frequency from 17.5 MHz to 13.5 MHz at different concentrations of glucose was used to construct a calibration curve, as shown in Figure 5A. Undiluted blood samples from three volunteers (*n* = 3) were then analyzed using the same setup. The analysis was performed three times for each sample and the time needed for conversion of AgCl to Ag was used to find the glucose concentration in whole blood from the calibration curve shown in Figure 5A. As shown in Figure 5B, the results of the proposed method showed good agreement with the standard method in whole blood glucose analysis. Table S5 summarizes the results obtained by the wireless biosensor cross-checked against the results of a commercially available glucometer. The results are encouraging; however, for building a practically competitive biosensor, the response time must be considerably shortened. As discussed above, the approach to exploiting the cathode layer comprised of Ag-AuNPs partially oxidized to the kΩ level will shorten the response time of the biosensor; however, full oxidation of the transduction layer to the MΩ level will generate more reproducible results when measuring glucose in whole blood samples. To avoid any inconsistency caused by the matrix effect, the concentration of glucose in whole blood was also determined using the standard addition method. Accordingly, blood samples were spiked with different concentrations of glucose in the range of 0–12 mM and were analyzed using the same biosensor setup. Figure 5C shows the plot of inverse response time versus different concentrations of glucose spiked to the whole blood. Extrapolation of the recorded responses to zero resulted in a blood glucose concentration of 5.88 mM, which was close to the concentration detected by the glucometer in the primary blood sample (5.3 mM). To show the efficiency of the bioanode part (enzyme biosensor) in whole blood analysis, the response times (*t*) found in the standard addition method were used to calculate the current (*I*) generated by the bioanode in successive addition of glucose through  . Here, the charge (*Q*) is proportional to the amount of AgNPs deposited on the cathode layer. The average *Q* for 11 electrodes prepared for this experiment was calculated to be 4441 ± 660 μC (*Q* was determined by integration of the area of *I* vs *t* when Ag was oxidizing to AgCl, Figure S11). Figure 5D demonstrates an increase in generated current together with increasing concentration of glucose in whole blood; the results are promising, showing that the efficiency of the enzyme biosensor is not affected by the other compounds/interferences during successive measurements in whole blood. Table S6 shows a comparison between the proposed sensor and previously reported wireless sensors for glucose measurement in whole blood or other real samples (e.g., interstitial fluid, sweat, urine, tears, and saliva). Some glucose sensors operating in passive and chip-less mode have been already reported; however, it is important to note that the sensor proposed in this work benefits from both a simple/cost-effective read-out system and efficient sensing mechanism based on direct electron transferring of enzyme glucose dehydrogenase.

## Conclusions

In this work, we present a novel design of glucose-to-resistance transduction, which enables simple battery-less and chip-less wireless detection of glucose. The biosensor is based on a glucose-sensing bioanode and a cathode layer comprised of AgNPs electrooxidized to AgCl. The cathode layer was inserted as a path of RF antenna wind. In this biosensor, the bioanode carried out direct electron transfer-based bioelectrocatalytic oxidation of glucose at a AuNP/4-ATP/GDH-modified electrode with concomitant reduction of AgCl to Ag on the cathode layer. We proved that the bioanode-cathode connection fulfilled a function of glucose-to-resistance transduction. The process of AgCl-to-Ag reduction, and thus, the detection of glucose, was wirelessly monitored by measuring the change of the reflection spectra, |*S*_11_|, of the biosensor antenna circuit. As a proof of concept, wireless detection of glucose in PBS and whole blood has been demonstrated. One of the limitations of the proposed concept is the relatively long time of biosensor response to clinically relevant glucose concentrations. The mitigation of this limitation requires a reproducible deposition of low amounts of silver nanoparticles in the cathode layer. The required deposition is hard to ensure by manual pipetting but might be achievable by modern inkjet or other thin-film technologies.

Analysis of equivalent circuits of this biosensor setup showed that the thin Ag/AgCl layer can be regarded as a general redox reaction-to-resistance transducer coupled to an RF antenna. Specifically, we demonstrate that, in the presence of glucose, the bioanode reduced AgCl in the cathodic transduction layer to metallic Ag. This process was confirmed by measurements of the transduction layer resistance, which changed from high (a few tenths of kΩ or MΩ) to low (usually below 10 Ω). Wireless detection of this resistance change was done by determining the characteristic frequency *f*_0_ and *Q*-factor of the antenna circuit by measuring the RF reflection spectra (|*S*_11_|). Specifically, the characteristic frequency of the wireless biosensor was 17.5 MHz when the cathodic transduction layer was comprised of AgCl. After the reduction of AgCl to Ag, the characteristic frequency of the wireless biosensor changed to 13.5 MHz. The time needed for this characteristic frequency transition (the response time) was dependent on the glucose concentration in the solution where the GDH-based bioanode and AgCl-containing transduction layer, as the cathode, were immersed to. The response time was shorter at higher concentrations of glucose. We also found that the response time can be tuned by manipulating the cathodic transduction layer, specifically, by admixing AuNPs and by deploying a lower or higher degree of the AgNP electrooxidation to AgCl. These experiments suggest that the transduction layer can be optimized to increase the sensitivity of this biosensor to glucose. Specifically, we experimentally showed that the transduction layer made from AgNP-AuNP mixture can provide a wireless response of the biosensor system to 4 mM glucose in less than 2 min.

Additionally, for understanding the mechanism of this battery-less and chip-less wireless biosensor, the equivalent circuit has been proposed and discussed. This analysis allowed one to conclude that, after the response to glucose, the biosensor wireless signal (|*S*_11_|) does not depend on the solution characteristics where the biosensor is immersed to, e.g., on the ionic strength of the solution. Future work is conducted to design biosensor tags responding to physiologically relevant glucose concentrations in less than 1 min.

## Experimental Section

### Materials

d-(+)-Glucose (≥99.5%), phosphate-buffered saline (PBS) tablets, silver nitrate (≥99.0%), gold(III) chloride trihydrate (≥99.9%), sodium citrate dihydrate (≥99.0%), l-ascorbic acid (≥99.0%), potassium chloride (≥99.0%), sulfuric acid (98%), methanol (≥99.9%), polyethylenimine (PEI, MW ≈ 25000 by LS), Nafion solution (5 wt %), and 4-aminothiophenol (4-ATP, 97%) were purchased from Sigma-Aldrich. Heme-containing, membrane-bound bacterial glucose dehydrogenase (GDH) from *Ewingella americana* was purified and characterized according to a previously published protocol.^39,45^ All aqueous solutions were prepared in ultrapure water (resistivity of 18.2 MΩ cm) purified by a Purelab Flex system (ELGA LabWater, High Wycombe, UK). For whole blood analysis, blood samples from healthy donors were collected in VACUETTE FC Mix tubes (Greiner Bio-One, Kremsmünster, Austria). The tubes contain a mixture of additives Na_2_EDTA, sodium fluoride, citric acid, and sodium citrate, which inhibits glycolysis and prevents coagulation. Blood collection for analysis with the biosensors was approved by the Swedish Ethical Board (approval number 2009/180).

### Synthesis of Silver and Gold Nanoparticles

Silver and gold nanoparticles were synthesized according to our previously reported protocols.^23^ For silver nanoparticles (AgNPs), ascorbic acid and sodium citrate were exploited as the reductant and capping agents, respectively. Briefly, 2 mL of sodium citrate aqueous solution (1%, w/v), 0.5 mL of silver nitrate (1%, w/v), and 1 mL of potassium chloride (8 mM) were consecutively added to 1.5 mL of distilled water under stirring conditions at room temperature. Concurrently, 95 mL of distilled water was heated to boiling and 100 μL of ascorbic acid aqueous solution (100 mM) was added. After 5 min, the mixture containing AgNO_3_ was added to the boiling ascorbic acid solution. The reaction mixture kept boiling for 1 h under stirring conditions, resulting in a change from a transparent solution to a yellow solution. The nanoparticle dispersion was left to cool down to room temperature and then kept in a fridge for subsequent characterization, processing, and applications. Obtained AgNPs had a 32 nm diameter (DLS measurement) and −39 mV zeta potential, and the concentration was 0.36 nM. Concentrated dispersion (approximately 0.18 μM) was obtained by centrifugation at 7000 rpm for 20 min. Characterization of AgNPs and their dispersion is described in Section S2.

Gold nanoparticles (AuNPs) were synthesized from gold(III) chloride trihydrate and trisodium citrate via the Turkevich method.^46^ Following this protocol, 50 mL of gold(III) chloride trihydrate aqueous solution (1 mM) was heated to 80 °C under stirring. Afterward, 10 mL of trisodium citrate (38.8 mM) was added to the above solution, which led to color changes from yellow to deep red. The nanoparticle dispersion was left to cool down to room temperature and then kept in the fridge for subsequent characterization, processing, and applications. Obtained AuNPs had a 16 nm diameter (DLS measurement) and −44 mV zeta potential, and the concentration was 2.1 nM. Concentrated dispersion (approximately 1 μM) was obtained by centrifugation at 7000 rpm for 20 min. Characterization of AuNPs and their dispersion is described in Section S2.

### Modification of the Screen-Printed Electrode (SPE) with Nanoparticles: Preparation of the Cathodic Transduction Layer

To prepare the cathodic transduction layer, gold SPEs (DRP-C223AT, Asturias, Spain) were partially covered with scotch tape, leaving an open area, approximately 2 mm long and 0.5 mm broad, connecting the working (W) and counter (C) electrodes. This area was filled with 0.5 μL (three layers) of AgNPs or AgNP and AuNP mixture. The nanoparticles were then dried at 50 °C. To prevent detachment of the particles along with enhancing the layer mechanical stability, 1 μL of Nafion solution (5 wt %) was drop-casted on the NPs.^47^ After all these modifications, the resistance across the NP-bridged working and counter electrodes was less than 10 Ω. Electrochemical conversion of AgNPs to AgCl on the SPE was carried out in PBS solutions using a three-electrode system in which external reference and counter electrodes were used together with the NP-modified SPE as a working electrode. AgNP oxidation to AgCl was conducted using an applied potential of 200 mV (vs SCE) for 120 s (referred to as full AgNP oxidation) or 70 mV for 12 s (referred to as partial AgNP oxidation). The SPEs encompassing the AgCl cathodic transduction layer were then thoroughly rinsed with water and kept dry. The transduction layer thickness, composition, and morphology were characterized by a number of surface techniques such as scanning electron microscopy, electrochemical impedance spectroscopy, etc. All characterization results are summarized in Sections S2, S4, and S5.

### Preparation of the Glucose Dehydrogenase-Modified Electrodes: Fabrication of the Bioanode

To make a glucose-sensing bioanode, glucose dehydrogenase (GDH) was immobilized on several types of electrodes, specifically, a gold disc electrode (GE, BASi, West Lafayette, USA), glassy carbon electrode (GCE), or a part of the gold counter electrode of SPE with surface areas of 0.02, 0.07, and 0.02 cm^2^, respectively. GE and GCE were polished on 1.0 μm and 0.1 μm alumina powders and rinsed with ultrapure water. Continuous cycling in 0.5 M sulfuric acid solutions was carried out for electrochemical cleaning of the electrodes in the applied potential range of −0.4 to 1.4 V (vs SCE) with the potential scan rate of 100 mV s^–1^. Following this procedure, a positively charged surface of glassy carbon electrode (GCE/PEI) was prepared by immersing the electrode into the 1 mg mL^–1^ solution of PEI in water for 2 min. Afterward, 3 μL of concentrated AuNPs was drop-casted on the gold electrode (GE) or GCE/PEI or on a part of the counter electrode of SPE and left to dry at room temperature. A self-assembled monolayer (SAM) of 4-aminothiophenol (4-ATP) on the surface-confined AuNPs was established by the incubation of the electrodes in 2 mM 4-ATP in nitrogen-saturated methanol solution for 2 h. The SAM-modified surfaces were further drop-covered with 3 μL of GDH (34 mg mL^–1^) and left for 2 h for the enzyme immobilization under humid conditions. Following these steps, two types of biosensors identified as GCE/PEI/AuNPs/4-ATP/GDH and GE/AuNPs/4-ATP/GDH were prepared, rinsed with PBS, and kept in the fridge at 4 °C in the same solution. GCE/PEI/AuNPs/4-ATP/GDH stands for glassy carbon electrode consecutively modified with polyethylene imine, AuNPs, 4-aminothioplenol, and finally, GDH enzyme. Similarly, GE/AuNPs/4-ATP/GDH stands for a plane gold electrode modified with AuNPs, 4-ATP, and GDH. To evaluate the bioelectrocatalytic properties of the GDH-modified electrodes, cyclic voltammograms were recorded in PBS solution in the presence and absence of glucose at the scan rate of 1 mV s^–1^. The cyclic voltammograms (Figure 1A, main text) were recorded within the potential range of −0.3 to 0.35 V (vs SCE) using an Ivium potentiostat in a three-electrode electrochemical cell. A platinum wire and SCE were exploited as auxiliary and reference electrodes, respectively.

### Measurements of Bioanode-Driven Reduction of AgCl to Ag on the Cathode

To realize an enzyme-catalyzed glucose-to-resistance transduction, the GDH-based bioanode was connected to the SPE hosting two electrodes, which were bridged by a AgCl layer. Specifically, the AgCl layer bridged working and counter electrodes and the bioanode was connected to the counter electrode. The described setup was immersed in PBS, and the current flowing between two electrodes of the SPE, across the AgCl bridge, was measured by a potentiostat in chronoamperometric mode with 5 mV applied DC voltage (Figure 1C, main text). The use of a multimeter for measuring the resistance of the layer was refused due to the risk of invoking electrochemical reactions; multimeters usually use applied voltage higher than 1 V for measurements of resistance. To achieve the bioanode-driven reduction of the AgCl bridge, a certain concentration of glucose was added to the measurement cell containing PBS. The current measurements were then used to calculate the resistance of the transduction layer using Ohm’s law (example of data in Figure 1D, main text).

### Measurements of Glucose in Whole Blood

The application of the proposed glucose biosensor in whole blood was tested using two different methods: calibration curve method and standard addition method. For the calibration curve method, SPEs modified with the bioanode and cathode layer (Ag-AuNPs oxidized to MΩ level) were prepared and connected to the tag antenna, as shown in Scheme 1. Following this step, standard solutions of glucose with the concentration range of 4–14 mM in PBS were analyzed on the SPE and the |*S*_11_| characteristic curve of the biosensor during conversion of AgCl (*f*_0_ = 17.5 MHz) to Ag (*f*_0_ = 13.5 MHz) was recorded wirelessly. The time needed for the transition of *f*_0_ was considered as the biosensor response (response time) and was used to construct a calibration plot, as shown in Figure 5A. With the same setup, 50 μL of unmodified blood samples, from three volunteers, was dropped on the SPE and the time needed for conversion of AgCl to Ag was used to find the glucose concentration in whole blood. The efficiency of the biosensor in whole blood analysis was also validated using a commercially available glucometer (HemoCue Glucose 201 RT system, Ängelholm, Sweden), which is known for its high accuracy in the diagnosis and screening of diabetes. For the standard addition method, blood samples were spiked with different concentrations of glucose in the range of 0–12 mM. Following this step, 50 μL of each blood sample, spiked with a specified concentration of glucose, was dropped on the SPE and the time for transition in characteristic frequency from 17.5 MHz to 13 MHz was recorded for each sample (concentration). The calibration curve obtained using blood was extrapolated to zero of the *y*-axis to find the glucose concentration in the primary blood sample.

### Wireless Measurement of AgCl-to-Ag Conversion on the Cathode: Recording of |*S*_11_| Characteristic

A simple vector network analyzer DG8-SAQ VNA (SDR-Kits, Melksham, UK) serving as a wireless radio-frequency (RF) antenna reader was used. The reader was equipped with a homemade copper circular antenna (diameter, 4 cm; four loops) connected to the TX port of a VNA. An NFC tag (Smartrac, 13.56 MHz tag) was exploited as an RF antenna of the biosensor and thus enabled wireless monitoring of biosensing reaction. To couple the SPE, modified with a Ag/AgCl-based transduction layer, into the tag antenna, 5 mm of the antenna path was cut out. The produced path ends were connected to the working and counter electrodes on the SPE bridged by the AgCl layer (a photo of the setup is demonstrated in Figure S12). For all reported wireless measurements, the reader and the tag antennas were kept at a fixed distance of 1 cm. During the wireless measurements, the magnitude of the reflection parameter |*S*_11_| was monitored continuously in the frequency range of 3–32 MHz. The magnitude of |*S*_11_| is defined by the following relation:
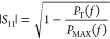
where *P*_T_ is the power transmitted to the tag antenna at a certain frequency, and *P*_MAX_ is the maximum achievable transmitted power. The |*S*_11_| depended on the frequency of the electromagnetic field created by the antenna of the reader. This dependence was used to determine the characteristic (resonance) frequency of the tag antenna circuit comprised of the tag antenna coupled to the SPE that hosted the transduction layer.

## Supporting Information Available

Characterization of AgNPs and AuNPs and their dispersions; resistance measurement of AgNPs or AgNP-AuNP layers during the electrochemical oxidation and reduction; characterization of the transduction layer by different techniques, SEM and EDX; characterization of AgNP- and AgCl-containing layers by electrochemical impedance spectroscopy; characterization of GDH-modified electrodes; repeatability of the wireless biosensor response; description of the equivalent electrical circuit of the chip-less wireless biosensor; whole blood sample analysis using the proposed wireless biosensor; characteristics of the proposed setup for wireless measurement of glucose (PDF)
