# Supplementary material for: Glucose-to-Resistor Transduction Integrated into a Radio-Frequency Antenna for Chip-less and Battery-less Wireless Sensing
Source: ACS Sens. 2022 Apr 7;7(4):1222–34. doi: 10.1021/acssensors.2c00394 (PMC9040053; doi:10.1021/acssensors.2c00394)
Supplement: Supplementary file 1 — se2c00394_si_001.pdf [file se2c00394_si_001.pdf]

Supporting Information

Glucose-to-resistor transduction integrated into RF antenna for chip-less and battery-less wireless sensing

Atefeh Shafaat[1,2], Rokas Žalnėravičius[3], Dalius Ratautas[4,5], Marius Dagys[4], Rolandas Meškys[4], Rasa Rutkienė[4], Juan Francisco Gonzalez-Martinez[1,2] , Jessica Neilands[6], Sebastian Björklund[1,2], Javier Sotres[1,2], and Tautgirdas Ruzgas[1,2]*

[1]Department of Biomedical Science, Faculty of Health and Society, Malmö University, 205 06 Malmö, Sweden
[2]Biofilms - Research Center for Biointerfaces, Malmö University, 205 06 Malmö, Sweden
[3]State Research Institute, Centre for Physical Sciences and Technology, Saulėtekio av. 3, LT-10257 Vilnius, Lithuania
[4]Institute of Biochemistry, Life Sciences Centre, Vilnius University, Saulėtekio al. 7, LT-10223, Vilnius, Lithuania
[5]Faculty of Fundamental Sciences, Vilnius Gediminas Technical University, Saulėtekio al. 11, LT-10223, Vilnius, Lithuania
[6]Department of Oral Biology, Faculty of Odontology, Malmö University, 205 06 Malmö, Sweden

* Corresponding authors: Tautgirdas Ruzgas (tautgirdas.ruzgas@mau.se)

Table of contents:

S1. Characterisation of AgNPs and AuNPs and their dispersions
S2. Resistance measurement of AgNPs or AgNP-AuNP layers during the electrochemical oxidation and reduction
S3. Characterization of the transduction layer by different techniques: SEM and EDX
S4. Characterisation of AgNP and AgCl containing layers by electrochemical impedance spectroscopy
S5. Characterisation of GDH modified electrodes
S6. Repeatability of the wireless biosensor response
S7. Description of equivalent electrical circuit of the chip-less wireless biosensor
S8. Whole blood sample analysis using the proposed wireless biosensor
S9. Characteristics of the proposed setup for wireless measurement of glucose

S1. Characterisation of AgNPs and AuNPs and their dispersions

By considering the fact that, e.g., the reduction potential of NPs might be affected by their characteristics such as size [1-3], different techniques were employed for characterization of synthesised NPs. Freshly prepared AgNP and AuNP dispersions were of yellow and red colour, respectively. The NPs were studied by transmission electron microscopy (TEM), dynamic light scattering (DLS) and small-angle X-ray scattering (SAXS) to assess their sizes and size distribution as well as zeta potential.

The morphology and size distribution of the nanoparticles were visualised by transmission electron microscope (TEM) FEI Tecnai F20 X-TWIN with a field emission gun. For this purpose, AuNPs and AgNPs were dispersed in ethanol and drop-casted onto carbon-coated nickel grid and allowed to dry. TEM images were recorded using Gatan Orius CCD camera in bright field mode at an acceleration voltage of 200 kV. ImageJ software was used to measure the mean particle diameter from the obtained images. The TEM images are shown in Fig. S1 and the values are summarised in Table S1.

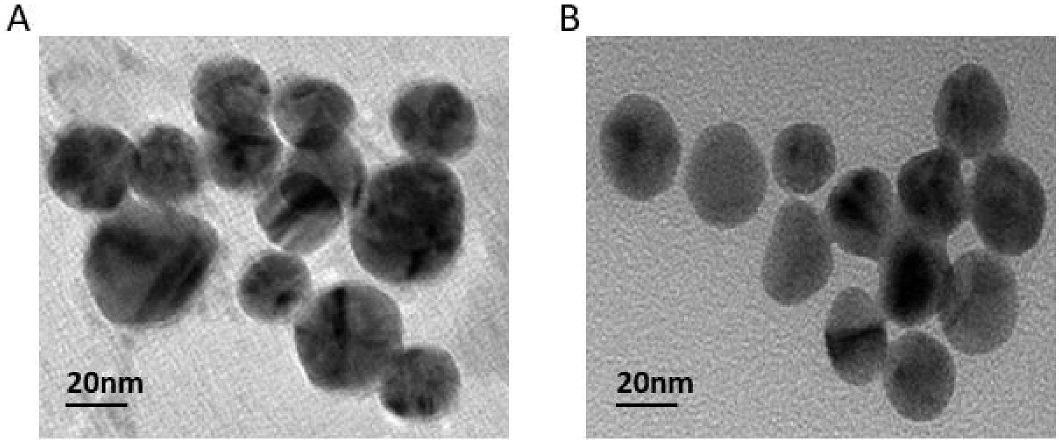

Fig. S1. TEM images of (A) AgNPs and (B) AuNPs, which were used in this study.

Size distribution, zeta potential and the concentration of the prepared AgNPs and AuNPs were determined by Zetasizer Ultra (ZSU5700, Malvern Panalytical, UK). The size distribution measurements were carried out in standard polystyrene (PS) cuvettes using multi-angle dynamic light scattering measurements (MADLS) in three different angels of backscatter, side scatter and forward scatter to give a global fitting for better resolution. The data are presented in Fig. S2A.

Zeta potential measurements were performed by using Zetasizer Ultra using the disposable folded capillary cells (DTS1080). On average, zeta potentials were found to be -39 mV and -44 mV for the negatively charged citrate-stabilized Ag and Au NPs. The original data on measurements of zeta potential are presented in Fig. S2B.

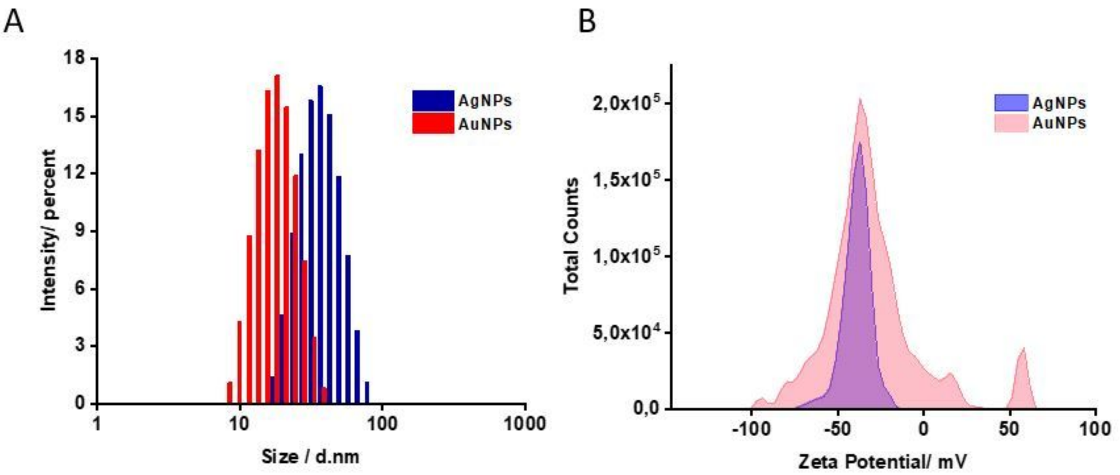

Fig. S2. Data collected using DLS instrument from Malvern. (A) Scattering intensity vs size and (B) zeta potential plots of the synthesized AgNPs and AuNPs.

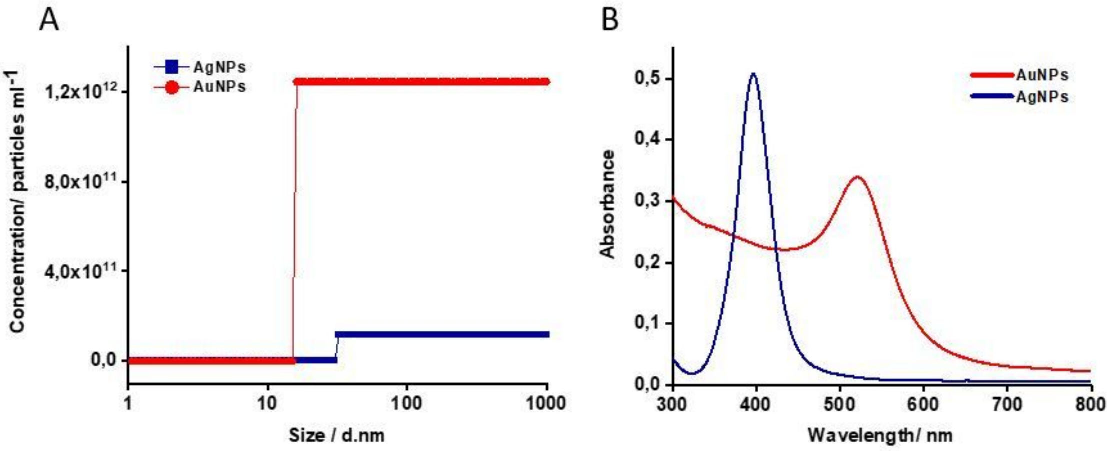

Fig. S3. (A) Concentration of the synthesized Ag and Au NP dispersions determined by using DLS instrument from Malvern. (B) LSPR spectra recorded for Ag and Au NPs.

Determination of the concentration, using the Zetasizer Ultra, was based on multi-angle dynamic light scattering measurements (MADLS). The technique transforms the intensity-weighted particle size distribution to the absolute concentration using the derived count rate and the optical properties of the nanoparticles and the suspension medium [4]. Before measuring the concentration of the colloidal nanoparticles, the background intensity of the dispersant (ultrapure water) was measured three times in order to subtract the background contribution. The results based on DLS measurements showed the concentration of $\approx 2 \times 10^{11}$ and $\approx 1 \times 10^{12}$ particles/ml for Ag and Au NPs, respectively (Fig. S3A). NP dispersions characterised by MADLS were also assessed by measurements of the localized surface plasmon resonance (LSPR) of the NPs using a UV-vis spectrophotometer (UV-1800 SHIMADZU, Kyoto, Japan). By assuming that the whole added amount of Ag and Au salts have been reduced and formed nanoparticles; the only attenuating products are nanoparticles, the concentration of AgNPs and AuNPs can be calculated using Beer Lambert's law [5, 6]. The extinction coefficients were taken to be 3.67×10^8 and 1.45×10^{10} M^{-1} cm^{-1} from the previously reported values at the wavelength of 520 nm and 406 nm for Au and Ag nanoparticles, respectively [7, 8]. The concentrations of dispersions estimated from these UV-vis experiments are presented in Table S1 and the corresponding absorption plots are shown Fig. S3B. The obtained concentrations from UV-vis measurements were found to be $\approx 5 \times 10^{11}$ and $\approx 5 \times 10^{12}$ particles/ml for Ag and Au NPs which were consistent with the one calculated by Zetasizer Ultra.

Table S1. Characteristics of Ag and Au NPs and their dispersions determined by different techniques/methods.

**Method**	**d $_{AgNPs}$/nm**	**d $_{AuNPs}$/nm**
MADLS	32,2 ± 1,6	16,1 ± 0,8
SAXS	25 ± 14	12 ± 5
TEM	16,8 ± 3,6	10,6 ± 0,9
**Method**	**C $_{AgNPs/}$ particles/ml**	**C $_{AuNPs/}$ particles/ml**
MADLS	$2,2 \times 10^{11} \pm 0,6$	$1,3 \times 10^{12} \pm 0,2$
UV-vis	$5,0 \times 10^{11} \pm 4,1$	$5,7 \times 10^{12} \pm 0,9$
**Method**	**ζ-potential $_{AgNPs}$/mV**	**ζ-potential $_{AuNPs}$/mV**
Zeta potential	-38,6 ± 2,0	-44,4 ± 2,1

To probe possible particle aggregation Small-angle X-ray Scattering (SAXS) data were collected using a XEUSS 3.0 instrument from Xenocs (Grenoble, France) with a photon energy E = 8 keV, using the Cu Kα radiation (λ = 1.54 Å). The signal was collected using a Pilatus 300K detector (Dectris). All the experiments were performed in vacuum at room temperature. The samples used for the experiment were: AuNP dispersion and AgNP dispersions were in water. All the samples were filled in 80 mm long borosilicate glass capillaries (WJM-Glass, Berlin, Germany) with the outside diameter of 1.5 mm and wall thickness of 0.01 mm. Standard corrections were done using XSact software from Xenocs [9] and the fits were done with SASView [10] to the dilute sphere model [11], so the final curves, shown in Fig. S4A, represents the result of the absolute intensity after subtraction of the solvent (water). The good agreement with the dilute sphere model and the linear behavior of the Guinier law fit at low Q values shows that there was no aggregation of nanoparticles.

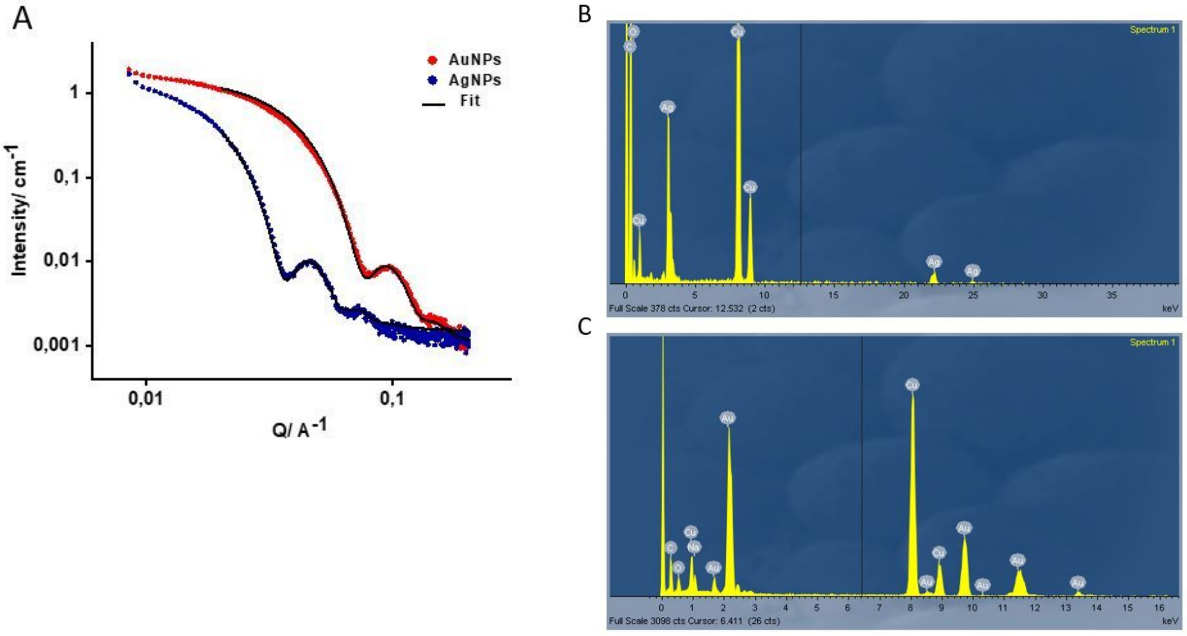

Fig. S4. (A) Scattering intensity profiles of measured SAXS data for as-synthesized silver and gold nanoparticles. (B, C) EDX spectrum of the synthesized (B) Ag and (C) Au NPs.

The elemental analysis of the Ag and Au NPs was also performed using the EDX (Energy-dispersive X-ray spectroscopy) on TEM and the results confirmed the high purity of the synthesized particles (Fig. S4). EDX spectrum of AgNPs shows a strong signal at 3 keV which is the main characteristic peak for AgNPs (Fig. S4B). The other peaks around 22 keV and 25 keV also correspond to the binding energies

of AgNPs [12, 13]. For AuNPs, the spectrum indicates a strong peak at 2.2 keV (Fig. S4C). There are some characteristic peaks at 8.5, 9.8 and 11.5 keV which are also relevant to metallic AuNPs [13, 14]. In EDX spectrum of both Ag and Au NPs some secondary crystals can be observed which are representative of sodium, carbon and oxygen coming from citrate groups. It should be mentioned that the copper peaks observed in both spectra correspond to the TEM grid holder.

S2. Resistance measurement of AgNPs or AgNP-AuNP layers during the electrochemical oxidation and reduction

In order to prove that AgNP or AgNP-AuNP comprised layer enables not only glucose-to-resistance transduction, but a more general redox-reaction-to-resistance transduction, the resistance of the layer made from AgNPs or AgNP-AuNP was measured during its electrochemical oxidation and reduction (Ag/AgCl conversion). For this, two potentiostats were connected to the SPE containing the transduction layer. One potentiostat was used to run cyclic voltammetry in three electrode mode with the SPE as working and external reference and counter electrodes. The applied potential ranged from -0.3 to +0.3 V and the scan rate was 10 mV s^{-1}. The other potentiostat was used to run chronoamperometry by applying a constant potential of 5 mV between the working and the counter electrodes on the SPE bridged by NP layer. The measured current by chronoamperometry was used to calculate the resistance of the layer in accordance to the Ohm's low. The connections of electrodes and potentiostats are illustrated in Fig. S5.

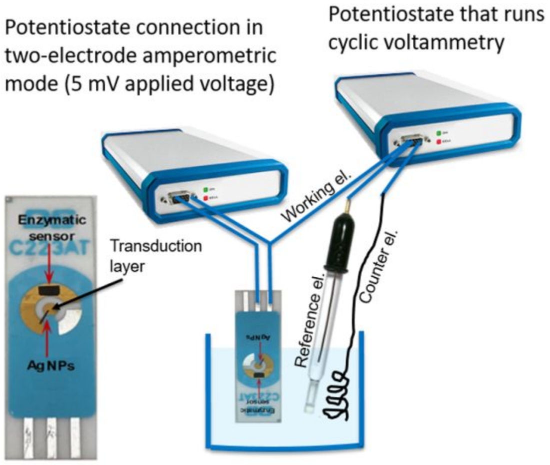

Fig. S5. Illustration of potentiostat-electrode connections for measurements of current that flow through the AgNP-AgCl containing transduction layer. Potentiostat in amperometric mode measures current at applied 5 mV DC potential. The current values are used to calculate the resistance of the transduction layer. The potentiostat that runs CV performs electrochemical Ag/AgCl redox conversion. The transduction layer containing AgNPs bridges two electrodes on SPE.

S3. Characterization of the transduction layer by different techniques: SEM and EDX

After electrooxidation of deposited NPs, AgNPs (100 %) or AgNP-AuNP mixture (80:20 %), the transduction layers were inspected by SEM. Fig. S6a and S6b show the SEM images of deposited AgNPs after electrooxidation. It is clear that the deposited layer is uneven/nonhomogeneous. As can be seen from Fig. S6b, AgNPs after electrooxidation form ≈ 300-900 nm different-shaped clumps. This is most probably due to AgNPs conversion to AgCl particles during electrooxidation. As confirmed by EDX spectra, the total amount of Cl was found to be ≈ 42 atomic percentage (Fig. S6c). Following these studies, fresh NP layer (before oxidation) was also evaluated by SEM (Fig. S6f) and the obtained results confirmed our hypothesis that the changes in morphology is caused by the electrooxidation of Ag to AgCl in PBS solution. Besides that, as shown in Fig. S6b and S6e, some small holes in the Ag and Ag-Au films are observed, which indicates that the electrochemical oxidation process leads to the formation of a porous surface with different thickness. This was confirmed by performing cross-sectional SEM images (Insets in Fig. S6f). These results also showed that the thickness of Ag-Au film deposited on SPE is ≈ 2 μm. Further, it should be noted that the AgCl particles form an overall compact structure,

with some porosity. This AgCl structure bridged two electrodes on SPE as a transduction layer of relatively high resistance (as compared to the AgNPs layer).

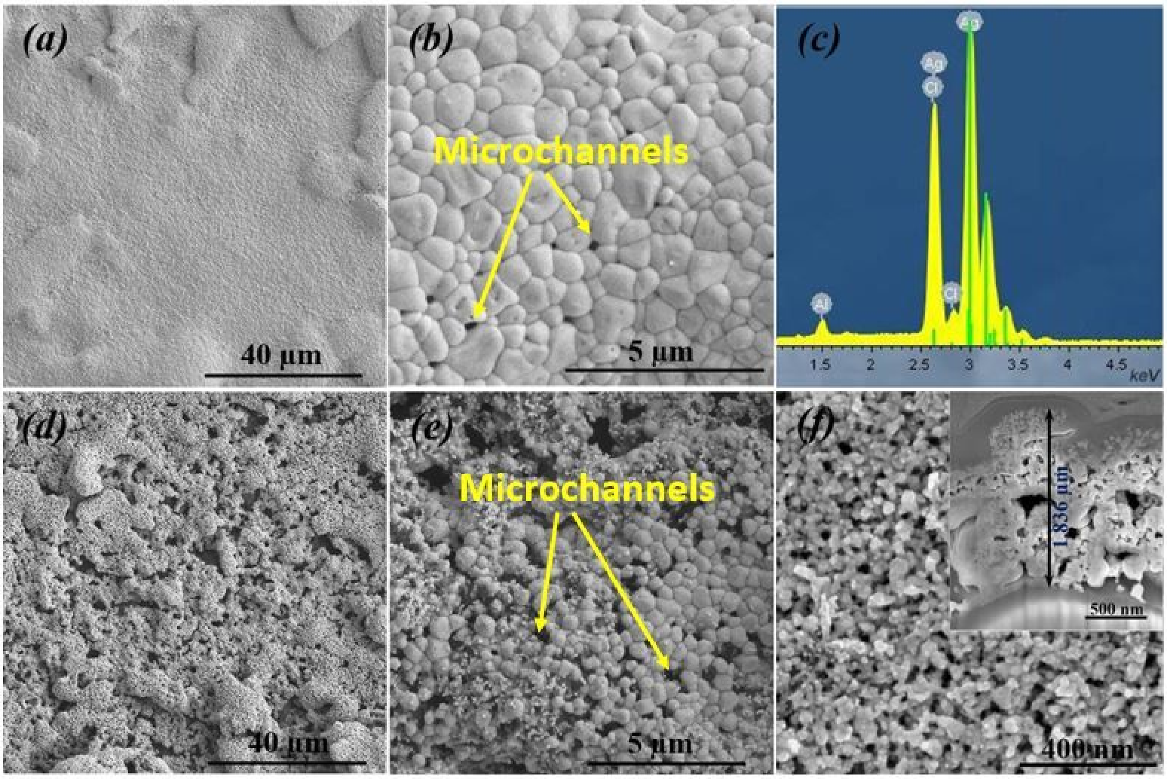

Fig. S6. (a and b) SEM images of oxidized (Ag→AgCl) layers of AgNPs and (d and e) Ag-AuNPs mixture on SPEs. (c) EDX spectrum taken from deposited layer. (f) SEM image of Ag-AuNPs mixture on SPE before oxidation. Insets: the cross-section SEM image of deposited Ag-AuNPs mixture on SPE.

S4. Characterisation of AgNP and AgCl containing layers by electrochemical impedance spectroscopy

In order to investigate the structure of the transduction layer resulting from Ag/AgCl conversion of AgNPs, electrochemical impedance spectroscopy (EIS) was conducted on SPE modified with AgCl-AuNPs in PBS solution at different AC voltage amplitude. The AC amplitudes ranged from 5 to 200 mV with the DC applied potential of 0.0 V. The EIS was run in the frequency range of 10 MHz to 0.1 Hz.

Interpretation of the EIS data accounted for porous structure (Fig. S7A) and were fitted with the electrochemical circuit illustrated in Fig. S7B. The EIS data can be explained by the presence of two layers on the electrode surface; the porous AgCl-AuNPs layer exposed to the electrolyte solution grown by electrochemical oxidation and the thin layer of Ag-AuNPs (Fig. S7A). Due to the surface inhomogeneity (rough and porous), the capacitive element (C_{dl}) does not show the ideal properties and accordingly was replaced by the constant phase element (Q) [15, 16]. R_s is the resistance of the electrolyte solution (PBS solution). R_1 and Q_1 are the resistance and constant phase element of the electrochemically grown AgCl-AuNPs layer exposed to the electrolyte. These characteristics depend on the defective structure or porosity of the layer [17]. On the other side, R_2 and Q_2 are the resistance and constant phase element of the layer of Ag-AuNPs deposited on the substrate.

The values of the proposed equivalent circuit obtained by fitting the experimental results shown in Fig. S7C and S7D are summarised in Table S2. The value of Q_1 is lower if compared to Q_2 which could be related to the higher thickness of the AgCl-AuNPs layer compared to the layer of Ag-AuNPs. The porous

structure of AgCl-AuNPs layer leads to the lower charge transfer resistance, measured for this layer (R_1 compared to R_2 values). It is also worth noting that by increasing the AC voltage amplitude from 5 to 200 mV, the values of charge transfer resistance for AgCl-AuNPs layer (R_1) decreases (Fig. S7C and S7D). This indicates the reduction of AgCl to Ag by the applied AC voltage, which can be modelled by the presence of a duplex layer on the substrate, as mentioned above. The present results describe a simple modelling of the AgCl to Ag conversion at low frequencies. However, association or correlation of these data to higher MHz frequencies, which are used in reading the RF antenna, is not easy to make. Though to consider possible correlations might be important for understanding RF effect on Ag/AgCl reactions.

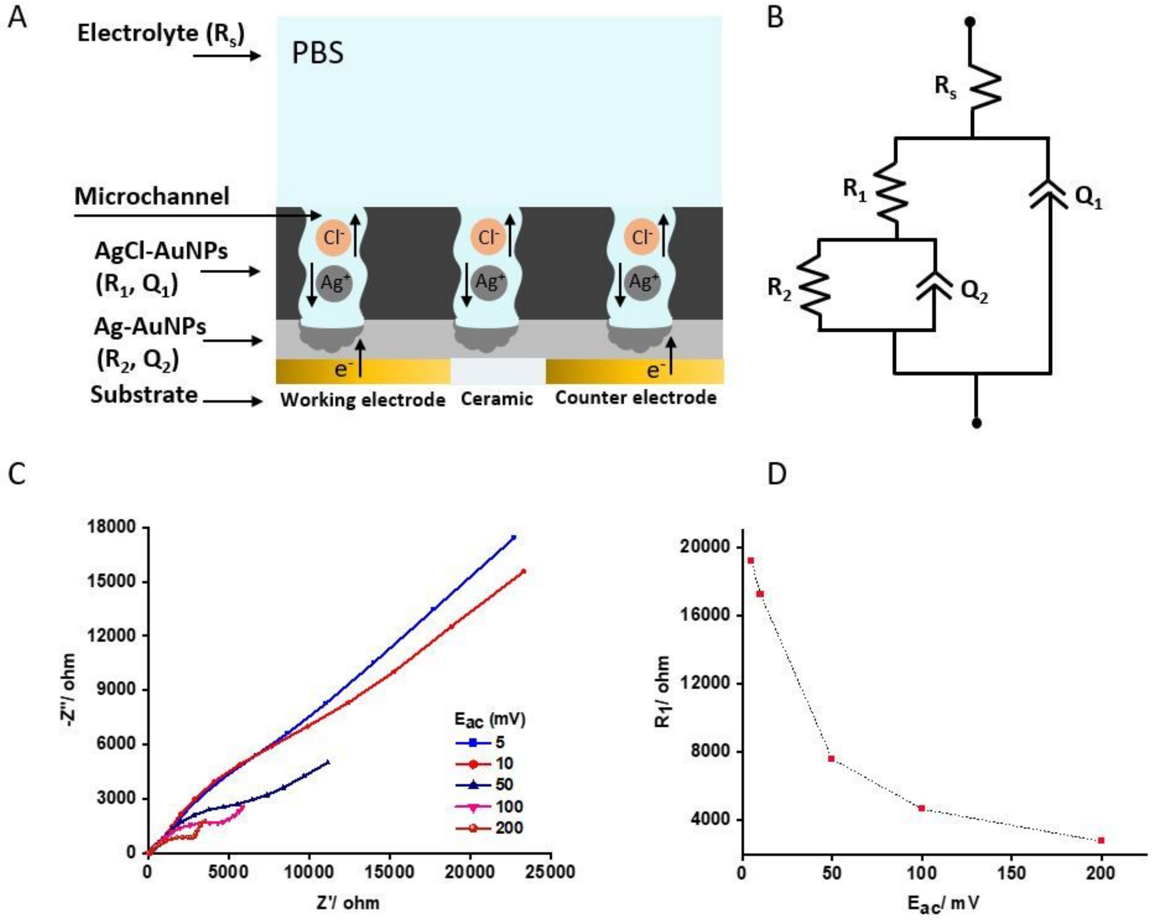

Fig. S7. (A) The proposed duplex layer on the substrate and (B) Equivalent circuit for signal modelling by EIS. (C) Nyquist plots and (D) changes in R_1 numerical values during impedance measurement on the screen-printed electrode modified with AgCl-AuNPs in PBS solution at the AC voltage amplitude of 5-200 mV and DC applied potential of 0.0 V

Table S2. The values of equivalent circuit elements, which describe impedance measurement using the SPE modified with AgCl-AuNPs layer bridging counter and working electrodes.

E_{ac} (mV)	5	10	50	100	200
R_s (ohm)	2,88E+02	2,22E+02	1,87E+02	1,37E+02	1,55E+02
R_2 (ohm)	5,26E+04	4,46E+04	1,15E+04	1,00E+04	6,90E+03
R_1 (ohm)	1,92E+04	1,72E+04	7,57E+03	4,65E+03	2,75E+03
Q_1	1,07E-05	1,05E-05	1,01E-05	8,89E-06	8,24E-06
Q_2	3,59E-05	5,26E-05	1,41E-04	3,42E-04	5,76E-04

S5. Characterisation of GDH modified electrodes

From comparison of CVs recorded with electrodes with 4-ATP immobilised GDH (Fig. 1A, main text) *vs* without 4-ATP immobilisation (Fig. S8A), it can be concluded that the maximum current density for 4-ATP modified electrodes is ≈ 15 times higher (0.75 mA cm^{-2} compared to 0.05 mA cm^{-2}). This confirms an essential role of 4-ATP for GDH immobilization in facile DET contact with the electrode surface. The immobilisation follows electrochemical transformation of 4-ATP. This is shown in CV for the electrode labelled as GCE/PEI/AuNPs/4-ATP. The CV shows a reversible redox process at the potential of 0.2V (vs. SCE) which indicated the formation of the redox active specie (Fig. S8B). According to the literature, 4-ATP can be oxidized at pH 7 to 4-mercapto-N-phenylquinone diimine (MPQD). This product is then hydrolysed rapidly to 4-mercapto-N-phenylquinone monoimine (MPQM) [18-20]. It has been reported that covalent attachment of enzyme on the surface of the MPQM-modified electrode occurs via a Schiff base formation between quinone groups of MPQM and the primary amino groups of glucose dehydrogenase. Since the redox potential of MPQM (≈ 0.2 V vs. SCE) is higher than the potential of the enzymatic reaction catalyzed by GDH (≈ -0.06 V vs. SCE), the MPQM acts as a crosslinking agent and not as a redox mediator. This explanation supports the statement about direct electron transfer coupling between the electrode and the GDH enzyme [18].

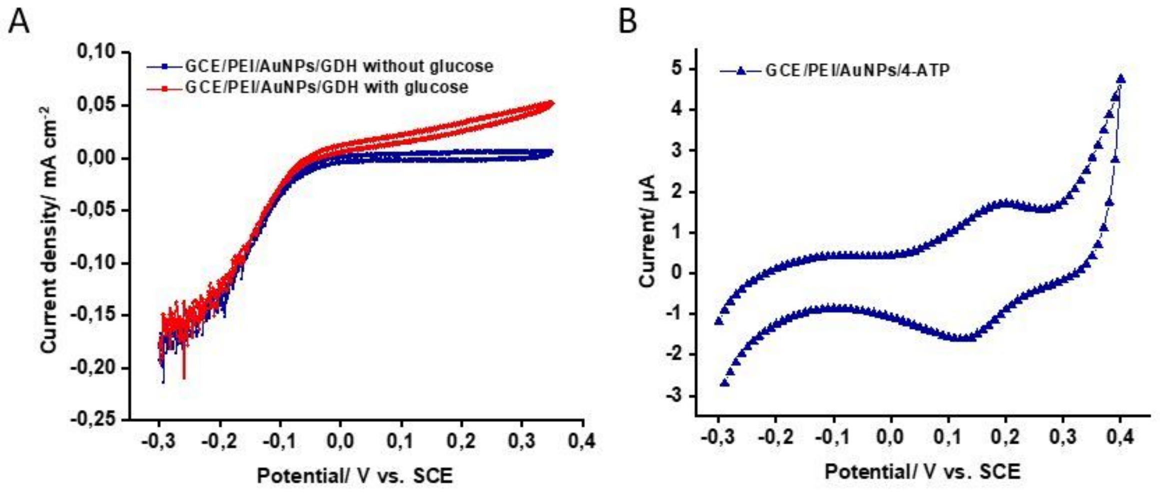

Fig. S8. (A) Cyclic voltammograms recorded with glucose dehydrogenase modified electrode, i.e., glucose sensing bioanode, constructed without 4-ATP modification, i.e., for electrode GCE/PEI/AuNPs/GDH. The CV measurements are done in PBS (pH 7.4), in the absence and presence of glucose (50 mM). Potential scan rate was 1 mV s^{-1}. (B) Cyclic voltammogram of 4-ATP modified electrode, i.e., GCE/PEI/AuNPs/4-ATP. The CV was recorded in PBS solution at the scan rate of 5 mV s^{-1}.

S6. Repeatability of the wireless biosensor response

Ten repeated responses to 6 mM of glucose have been recorded with the same SPE bridged with AuNP-AgNP layer which was electrooxidised to AuNP-AgCl transduction layer. Each measurement of glucose reduced AgCl to Ag. To prepare the transduction layer for the repeated measurement, after each response of the biosensor to glucose, the SPE with the transduction layer was place into PBS and the layer was electrooxidised to AgCl state by applying 70 mV for 12 seconds. After that a new measurement of glucose was carried out. The response times of the wireless biosensor obtained during these repeated measurements are shown in Fig. S9.

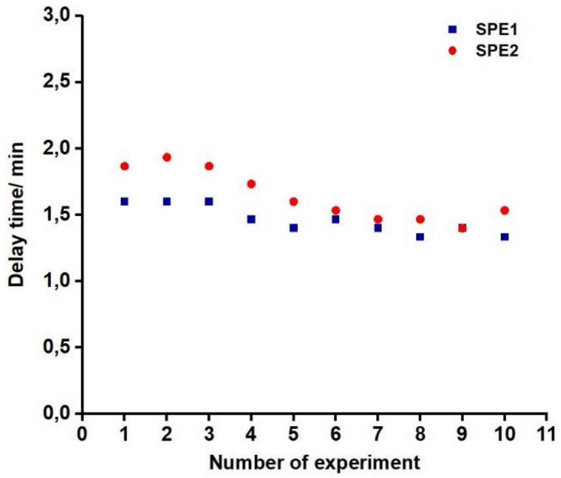

Fig. S9. The response time to 6 mM of glucose recorded with the biosensor tag. The same SPE modified with AuNP-AgNP mixture and oxidized to kΩ level was re-used. Data are from two independently prepared SPE. The GDH-based bioanode was connected to the cathodic transduction layer as a separate AuNP/4-ATP/GDH modified electrode (coupling is shown in Fig. 1C, main text).

S7. Description of equivalent electrical circuit of the chip-less wireless biosensor

The entire wireless biosensor is comprised of RF antenna connected to an SPE which hosts GDH based bioanode and AgCl based cathode layer, i.e., the bioanode-cathode connection which enables glucose-to-resistance transduction (see Fig. 4C, main text). It is important to note that only the cathode layer is included into the RF antenna circuit (tag antenna circuit), which is wirelessly addressed by the VNA based antenna reader. To better understand features of the wireless biosensor its antenna circuit is here discussed in term of equivalent circuit. To propose equivalent circuit for the biosensor tag design, function S11, and thus, f_0 and Q were determined with the SPE, containing AgCl or Ag transduction layer, immersed in the solution of different ionic strength (data in Table S3).

Table S3. Values of f_0 and Q determined for the tag, RF antenna with connected SPE (see Fig. 4C, main text). The SPE contained AgCl, or Ag comprised transduction layer specified as the material of transduction layer: AgCl or Ag. The SPE with transduction layer was immersed into the solution of different ionic strength (noted as I in mM) which was based on diluted or concentrated PBS.

		**Material of transduction layer**			
		**AgCl**		**Ag**	
**Media where the SPE with transduction layer is exposed to**	I, mM	$f_{0(AgCl)}$, MHz	$Q_{(AgCl)}$	$f_{0(Ag)}$, MHz	$Q_{(Ag)}$
Air	-	19.6708	18.2392	13.0345	11.69
Pure water	-	19.1261	17.9856	13.0345	11.69
PBS 100 times diluted	1.657	19.1261	16.00	13.0345	11.70
PBS 50 times diluted	3.314	19.1261	14.3937	13.0345	11.70
PBS 10 times diluted	16.57	19.027	9.30445	13.0345	11.70
PBS 5 times diluted	33.14	18.8784	7.02333	13.0345	11.70
PBS 2,5 times diluted	66.28	18.5318	4.90749	13.0345	11.70
PBS	165.7	16.8479	2.92171	13.0345	11.69
PBS 10 times concentrated	1657	13.1831	5.664	13.0345	11.69

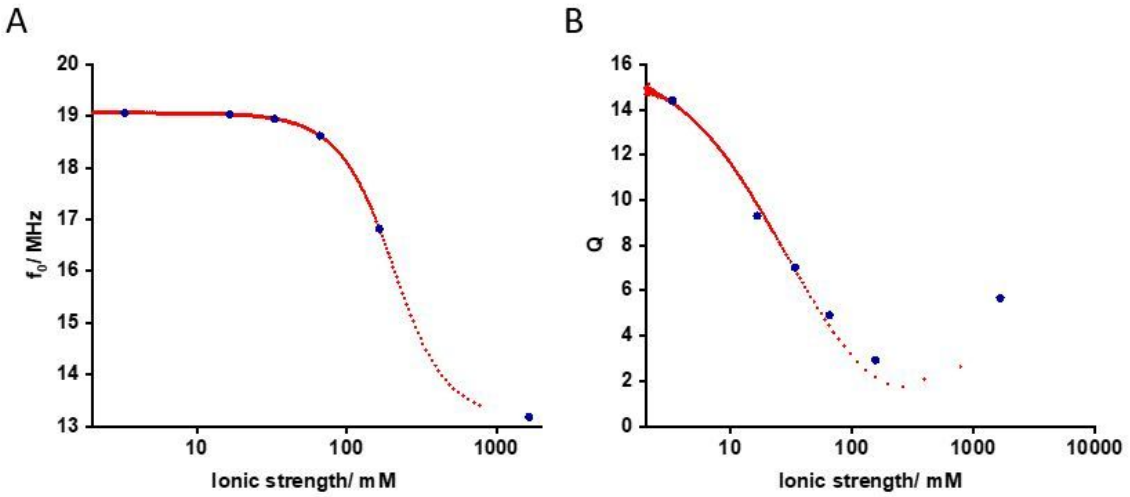

Fig. S10. (A) The characteristic frequency ($f_{0(AgCl)}$) and (B) Q-factor ($Q_{(AgCl)}$) of the antenna with a coupled SPE exposed to air or immersed into solution of different ionic strength (I, in mM). The SPE hosts a transduction layer comprised of AgCl. Equivalent circuit representing the antenna circuit is presented in Fig. 3D, main text, and discussed in Fig. 4C, main text.

Circuit C(c) in Fig. 4C represents a suggestion of the complete biosensor tag antenna circuit. This equivalent circuit was used to derive equations (Eq. S1, S2 and S3) for rigorous mathematical fitting of the experimental f_0 and Q dependencies on ionic strength (practically estimated by R2). The mathematically modelled best fits are shown in Fig. S10A and S10B.

The analytical equations used to calculate the f_0 and Q values are presented below.

$$Z(\omega) = R_1 + \frac{R_2}{1+\omega^2 R_2^2 C_2^2} + j\left(\omega L_1 - \frac{1}{\omega C_1} - \frac{\omega C_2 R_2^2}{1+\omega^2 R_2^2 C_2^2}\right) = Z_R + j\, Z_{im} \quad \text{Eq. S1}$$

Where $\omega = 2\pi f$ and R, L, C elements are defined in the circuit shown in Fig. 3 and Fig. 4, main text.

The condition for the characteristic frequency, $\omega_0 = 2\pi f_0$, is $Z_{im} = 0$. This leads to the following equation.

$$\omega_0 = \sqrt{2}\left(C_1L_1 - C_1C_2R_2^2 - C_2^2R_2^2 + \left(4C_1L_1C_2^2R_2^2 + \left(C_1L_1 - C_2(C_1+C_2)R_2^2\right)^2\right)^{\frac{1}{2}}\right)^{-\frac{1}{2}} \quad \text{Eq. S2}$$

The equation to find the bandwidth frequencies (power at Full Width Half Maximum, FWHM) can be expressed by the following equation:

$$|Z(\omega)|^2 = 2 \cdot Z(\omega_0) \quad \text{Eq. S3}$$

The Eq. S3 results in a sixth-degree Cardan polynomial of the type $P(\omega) = a\,\omega^6 + b\omega^4 + c\omega^2 + d$, whose roots can be found using radicals. From these roots, two are real positive solutions (ω_1,ω_2). These two solutions correspond with the limits of the bandwidth, which can be used to find the Q value:

$$(\omega_1,\omega_2) \Rightarrow Q = \frac{\omega_0}{|\omega_2 - \omega_1|} \quad \text{Eq. S4}$$

The equations have been written in Mathematica symbolic language and fitted to experimental f_0 and Q values. As can be seen (Fig. S10A and S10B) that the modelled curves relatively well describe experimentally obtained dependencies of f_0 and Q on the ionic strength of solution where the transduction layer comprised AgCl is immersed to. The values of the circuit elements derived from the fitting are presented in the main text and are as follows. For R_1, L_1, C_1, and C_2 the are equal to 0.0417 Ω, $5.61 \cdot 10^{-9}$ H, $2.62 \cdot 10^{-8}$ F, and $2.36 \cdot 10^{-8}$ F, respectively. Summarising, the fitting relatively well approximates the experimental data and, thus, confirms that the suggested equivalent circuit can be used for the representation of the antenna circuit of this novel biosensor tag. Table S4, additionally, presents

a list of simple equations, which support simple interpretation of the equivalent circuit of the biosensor tag antenna circuit.

Table S4. Summary of equations for estimating characteristic frequency ($f_{0(\mathrm{Ag})}$, $f_{0(\mathrm{AgCl})}$) and Q-factor ($Q_{(\mathrm{Ag})}$, $Q_{(\mathrm{AgCl})}$) of tag antenna with a coupled SPE exposed to air, immersed into H_2O and solution of different ionic strength. The SPE hosts a transduction layer comprised of Ag or AgCl. The table lists also experimental values of f_0 and Q obtained at specified conditions. Values R_1, R_2, L_1, C_1, and C_2 are elements of the circuit shown in Fig. 3D and Fig. 4C.

**State of the transduction layer, circuit C1-C4**	**f_0, MHz (formulas and experimental values)**	**Q (formulas and experimental values)**
Transduction layer: Ag.<br>Circuit: Fig. 4C, C(a).	$f_{0(Ag)} = \frac{1}{2\pi\sqrt{L_1C_1}}\left[\frac{Rad}{s}\right]$	$Q_{(Ag)} = \frac{1}{R_1}\sqrt{\frac{L_1}{C_1}}$
	13.03 MHz	11.7
Transduction layer: AgCl in 10xPBS.<br>Fig. 4C, C(b).	$f_{0(Ag)} \approx f_{0(AgCl),10xPBS}$<br>$f_{0(AgCl)} = \frac{1}{2\pi\sqrt{L_1C_1}}\left[\frac{Rad}{s}\right]$	$Q_{(AgCl)} = \frac{1}{R_1 + R_2}\sqrt{\frac{L_1}{C_1}}$
	13.18 MHz	5.66
Transduction layer: AgCl in H_2O or in air.<br>Fig. 4C, C(d).<br>$C_T = \frac{C_1C_2}{C_1 + C_2}$	$f_{0(AgCl),H_2O} \approx f_{0(AgCl),air}$<br>$f_{0(AgCl)} = \frac{1}{2\pi\sqrt{L_1C_T}}\left[\frac{Rad}{s}\right]$	$Q_{(AgCl),H_2O} \approx Q_{(AgCl),air}$<br>$Q_{(AgCl)} = \frac{1}{R_1}\sqrt{\frac{L_1}{C_T}}$
	19.13 MHz (in H_2O)<br>19.67 MHz (in air)	17.99 (in H_2O)<br>18.24 (in air)

S8. Whole blood sample analysis using the proposed wireless biosensor

Table S5. The result of comparison between the proposed wireless sensor and the standard method (glucometer) for glucose analysis in the whole blood samples.

**Samples**	**Glucose concentration/ Standard method (mM)**	**Mean (SD)**	**Glucose concentration/ Proposed biosensor (mM)**	**Mean (SD)**
**Sample 1**	5.3<br>5.0<br>5.0	5.1 (± 0.17)	5.72<br>4.74<br>5.07	5.18 (± 0.50)
**Sample 2**	5.8<br>6.1<br>6.4	6.1 (± 0.30)	5.14<br>5.96<br>6.19	5.76 (± 0.55)
**Sample 3**	4.9<br>5.1<br>4.8	4.9 (± 0.15)	6.03<br>5.28<br>6.28	5.86 (± 0.51)

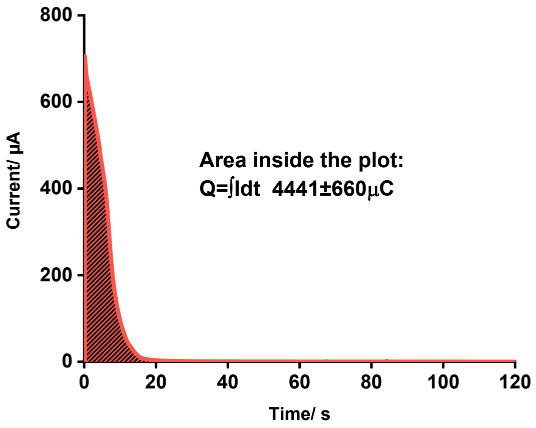

Fig. S11. Chronoamperogram shows the current-time dependence obtained during oxidation of cathode layer comprised of Ag-AuNPs to AgCl. The oxidation resulted into the layer resistance of MΩ level. The oxidation to AgCl in the transduction layer was achieved at 200 mV applied voltage for 120 s in PBS solution. Included is the average charge, needed to convert Ag to AgCl, found for 11 electrodes prepared for measurements of 11 different concentrations of glucose spiked in whole blood.

S9. Characteristics of the proposed setup for wireless measurement of glucose

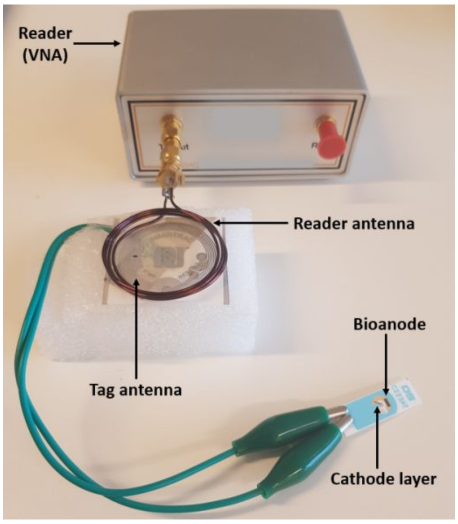

Fig. S12. A photograph of the setup including a vector network analyzer (VNA) equipped with a homemade copper circular antenna, NFC tag with 5 mm cut out of antenna wind for connecting gold SPE modified with bioanode and cathode layers.

Table S6. Comparison of the proposed sensor with the previously reported wireless sensors for glucose measurement.

Sensing mechanism	Linear range	Stability	System design	Wireless connection	Sampled from	Ref
GDH/direct	4-12 mM	30 days	Chip-less/Battery-less	Radio frequency	Blood	This work
GOx/mediated	1-30 mM	30 days	Integrated chip	Bluetooth	Interstitial fluid	21
GOx/mediated	1-10 mM	-	Integrated circuit	Integrated Wi-Fi module	Blood	22
GOx	0-5 mM	15 days	Integrated circuit	Bluetooth	Sweat	23
GDH/mediated	0-10 mM	-	Self-Powered	Bluetooth	Urine sugar	24
GOx/mediated	0-22 mM	-	Integrated circuit	Bluetooth	Interstitial fluid	25
non-enzymatic	5-20 mM	-	NFC chip	NFC	Peritoneal cavity	26
GOx	0-6 mM	-	Integrated circuit	Bluetooth	Sweat	27
non-enzymatic	0.5-2 mg/mL	-	Integrated circuit	Bluetooth	Sweat	28
non-enzymatic	0-32 mM	30 days	Integrated circuit	Bluetooth	Interstitial fluid	29
GOx/direct	50-400 µM	-	Integrated circuit	Bluetooth	Sweat	30
GOx	5-20 µM	-	NFC chip	NFC	Sweat	31
non-enzymatic	5 µM-0.25 mM	30 days	Integrated circuit	Bluetooth	Buffer	32
GOx	0.1 nM-10 mM	-	-	Microwave signal	Sweat	33
GOx/mediated	0-150 µM	-	Integrated circuit	Bluetooth	Sweat	34
GOx	3-25 mg/dL	-	Chip/Thin-film battery	Radio frequency	Human tears	35
PBA hydrogel	-	45 days	-	Radio frequency	Interstitial fluid	36
GOx/direct	0-300 µM	-	Integrated circuit	Bluetooth	Sweat	37
Glucose-sensitive hydrogel	0-2 mM and 8-10 mM	-	-	Radio frequency	Buffer	38
non-enzymatic	0-500 mg/dL	-	-	Radio frequency /Microwave	Interstitial fluid	39
GOx	36-360 mg/dL	-	Integrated microchip/Battery powered	Bluetooth/RFID	Interstitial fluid	40
GOx	0.1-1.4 mM	-	Integrated circuit	Bluetooth	Saliva	41
GOx/mediated	0-2.1 mM	-	-	Bluetooth	Buffer	42
GOx	0.5-50 mg/dL	14 days	Integrated circuit/Thin-film battery	Radio frequency	Sweat	43
non-enzymatic	10-500 mg/dL	-	-	Surface acoustic wave /Radio frequency	Buffer	44
GOx/mediated	-	-	Integrated circuit	Bluetooth	Human tears	45
GOx/mediated	0-9 mM	-	Integrated circuit	Wi-Fi-based microcontroller	Buffer	46
GOx/mediated	100-500 µm	-	NFC chip	NFC	Sweat	47
non-enzymatic	10-200 µM		Integrated circuit	Wi-Fi	Sweat	48
non-enzymatic	0.5 nM-10 mM	-	-	Bluetooth	Sweat	49
non-enzymatic	0-0.075 mol	-	-	Radio frequency	water	50
GOx/mediated	0.05-20 mM	-	NFC chip/Rechargeable battery	Bluetooth/NFC	Blood	51

References

1. Ivanova, O. S.; Zamborini, F. P., Size-dependent electrochemical oxidation of silver nanoparticles. *J Am Chem Soc* **2010,** *132* (1), 70-72.

2. Henglein, A., Physicochemical Properties of Small Metal Particles in Solution - Microelectrode Reactions, Chemisorption, Composite Metal Particles, and the Atom-to-Metal Transition. *Journal of Physical Chemistry* **1993,** *97* (21), 5457-5471.

3. Henglein, A., The reactivity of silver atoms in aqueous solutions (a γ-radiolysis study). *Berichte der Bunsengesellschaft für physikalische Chemie* **1977,** *81* (6), 556-561.

4. Austin, J.; Minelli, C.; Hamilton, D.; Wywijas, M.; Jones, H. J., Nanoparticle number concentration measurements by multi-angle dynamic light scattering. *Journal of Nanoparticle Research* **2020,** *22* (5), 1-15.

5. Rahman, S., Size and concentration analysis of gold nanoparticles with ultraviolet-visible spectroscopy. *Undergraduate Journal of Mathematical Modeling: One+ Two* **2016,** *7* (1), 2. DOI: 10.5038/2326-3652.7.1.4872.

6. Swinehart, D. F., The Beer-Lambert Law. *Journal of Chemical Education* **1962,** *39* (7), 333-335.

7. Aldrich, S., Gold nanoparticles: properties and applications. *Sigma-Aldrich, St. Louis, MO* **2015**.

8. Paramelle, D.; Sadovoy, A.; Gorelik, S.; Free, P.; Hobley, J.; Fernig, D. G., A rapid method to estimate the concentration of citrate capped silver nanoparticles from UV-visible light spectra. *Analyst* **2014,** *139* (19), 4855-4861.

9. Xenocs, "XSACT: X-ray Scattering Analysis and Calculation Tool." xsact.xenocs.com, 2021. SAXS & WAXS data analysis software — Version 2.4.

10. SASView, This work benefited from the use of the SasView application, originally developed under NSF award DMR-0520547. SasView contains code developed with funding from the European Union's Horizon 2020 research and innovation programme under the SINE2020 project, grant agreement No 654000.

11. Londoño, O. M.; Tancredi, P.; Rivas, P.; Muraca, D.; Socolovsky, L. M.; Knobel, M., *Small-angle X-ray scattering to analyze the morphological properties of nanoparticulated systems. In Handbook of materials characterization (pp. 37-75)*. Springer: Cham, 2018.

12. Guzman, M. G.; Dille, J.; Godet, S., Synthesis of silver nanoparticles by chemical reduction method and their antibacterial activity. *Int. J. Chem. Biomol. Eng.* **2009,** *2* (3), 104-111.

13. Singh, P.; Kim, Y. J.; Wang, C.; Mathiyalagan, R.; Yang, D. C., The development of a green approach for the biosynthesis of silver and gold nanoparticles by using Panax ginseng root extract, and their biological applications. *Artif Cells Nanomed Biotechnol* **2016,** *44* (4), 1150-1157.

14. Pan, C.; Fang, Y.; Wu, H.; Ahmad, M.; Luo, Z.; Li, Q.; Xie, J.; Yan, X.; Wu, L.; Wang, Z. L.; Zhu, J., Generating electricity from biofluid with a nanowire-based biofuel cell for self-powered nanodevices. *Adv Mater* **2010,** *22* (47), 5388-5392.

15. Jimenez-Morales, A.; Galvan, J.; Rodriguez, R.; De Damborenea, J., Electrochemical study of the corrosion behaviour of copper surfaces modified by nitrogen ion implantation. *Journal of applied electrochemistry* **1997,** *27* (5), 550-557.

16. Martini, E. M. A.; Muller, I. L., Characterization of the film formed on iron in borate solution by electrochemical impedance spectroscopy. *Corrosion Science* **2000,** *42* (3), 443-454.

17. Mahato, N.; Singh, M. M., Investigation of Passive Film Properties and Pitting Resistance of AISI 316 in Aqueous Ethanoic Acid Containing Chloride Ions using Electrochemical Impedance Spectroscopy(EIS). *Portugaliae Electrochimica Acta* **2011,** *29* (4), 233-251.

18. Ratautas, D.; Laurynėnas, A.; Dagys, M.; Marcinkevičienė, L.; Meškys, R.; Kulys, J., High current, low redox potential mediatorless bioanode based on gold nanoparticles and glucose dehydrogenase from Ewingella americana. *Electrochimica Acta* **2016,** *199*, 254-260.

19. Lukkari, J.; Kleemola, K.; Meretoja, M.; Ollonqvist, T.; Kankare, J., Electrochemical post-self-assembly transformation of 4-aminothiophenol monolayers on gold electrodes. *Langmuir* **1998,** *14* (7), 1705-1715.

20. Raj, C. R.; Kitamura, F.; Ohsaka, T., Electrochemical and in situ FTIR spectroscopic investigation on the electrochemical transformation of 4-aminothiophenol on a gold electrode in neutral solution. *Langmuir* **2001,** *17* (23), 7378-7386.

21. Jin, X.; Li, G.; Xu, T.; Su, L.; Yan, D.; Zhang, X., Fully integrated flexible biosensor for wearable continuous glucose monitoring. *Biosensors and Bioelectronics* **2022,** *196*, 113760-113764.
22. Escalona-Villalpando, R. A.; Sandoval-García, A.; Espinosa L, J. R.; Miranda-Silva, M. G.; Arriaga, L. G.; Minteer, S. D.; Ledesma-García, J., A self-powered glucose biosensor device based on microfluidics using human blood. *Journal of Power Sources* **2021,** *515*, 230631-230638.
23. Li, G.; Hao, J.; Li, W.; Ma, F.; Ma, T.; Gao, W.; Yu, Y.; Wen, D., Integrating Highly Porous and Flexible Au Hydrogels with Soft-MEMS Technologies for High-Performance Wearable Biosensing. *Analytical Chemistry* **2021,** *93* (42), 14068-14075.
24. Shitanda, I.; Fujimura, Y.; Takarada, T.; Suzuki, R.; Aikawa, T.; Itagaki, M.; Tsujimura, S., Self-Powered Diaper Sensor with Wireless Transmitter Powered by Paper-Based Biofuel Cell with Urine Glucose as Fuel. *ACS Sensors* **2021,** *6* (9), 3409-3415.
25. De la Paz, E.; Barfidokht, A.; Rios, S.; Brown, C.; Chao, E.; Wang, J., Extended Noninvasive Glucose Monitoring in the Interstitial Fluid Using an Epidermal Biosensing Patch. *Analytical Chemistry* **2021,** *93* (37), 12767-12775.
26. Xu, J.; Cheng, C.; Li, X.; Lu, Y.; Hu, S.; Liu, G.; Zhu, L.; Wang, N.; Wang, L.; Cheng, P.; Su, B.; Liu, Q., Implantable platinum nanotree microelectrode with a battery-free electrochemical patch for peritoneal carcinomatosis monitoring. *Biosensors and Bioelectronics* **2021,** *185*, 113265-113271.
27. De Pascali, C.; Francioso, L.; Giampetruzzi, L.; Rescio, G.; Signore, M. A.; Leone, A.; Siciliano, P., Modeling, Fabrication and Integration of Wearable Smart Sensors in a Monitoring Platform for Diabetic Patients. *Sensors* **2021,** *21* (5), 1847-1867.
28. Shi, W.; Li, Q.; Zhang, Y.; Liu, K.; Huang, X.; Yang, X.; Ran, Y.; Li, Y.; Guo, Y.; Liu, Y., Enabling the aqueous solution sensing of skin-conformable organic field-effect transistor using an amphiphilic molecule. *Applied Materials Today* **2022**, 101275-101284.
29. Lei, L.; Zhao, C.; Zhu, X.; Yuan, S.; Dong, X.; Zuo, Y.; Liu, H., Nonenzymatic Electrochemical Sensor for Wearable Interstitial Fluid Glucose Monitoring. *Electroanalysis* **2022,** *34* (2), 415-422.
30. Lin, H.; Tan, J.; Zhu, J.; Lin, S.; Zhao, Y.; Yu, W.; Hojaiji, H.; Wang, B.; Yang, S.; Cheng, X.; Wang, Z.; Tang, E.; Yeung, C.; Emaminejad, S., A programmable epidermal microfluidic valving system for wearable biofluid management and contextual biomarker analysis. *Nature Communications* **2020,** *11* (1), 4405-4416.
31. Kim, S.; Lee, B.; Reeder, J. T.; Seo, S. H.; Lee, S.-U.; Hourlier-Fargette, A.; Shin, J.; Sekine, Y.; Jeong, H.; Oh, Y. S.; Aranyosi, A. J.; Lee, S. P.; Model, J. B.; Lee, G.; Seo, M.-H.; Kwak, S. S.; Jo, S.; Park, G.; Han, S.; Park, I.; Jung, H.-I.; Ghaffari, R.; Koo, J.; Braun, P. V.; Rogers, J. A., Soft, skin-interfaced microfluidic systems with integrated immunoassays, fluorometric sensors, and impedance measurement capabilities. *Proceedings of the National Academy of Sciences* **2020,** *117* (45), 27906-27915.
32. Jo, H. J.; Shit, A.; Jhon, H. S.; Park, S. Y., Highly sensitive non-enzymatic wireless glucose sensor based on Ni–Co oxide nanoneedle-anchored polymer dots. *Journal of Industrial and Engineering Chemistry* **2020,** *89*, 485-493.
33. Xue, Q.; Li, Z.; Wang, Q.; Pan, W.; Chang, Y.; Duan, X., Nanostrip flexible microwave enzymatic biosensor for noninvasive epidermal glucose sensing. *Nanoscale Horizons* **2020,** *5* (6), 934-943.
34. Yu, Y.; Nassar, J.; Xu, C.; Min, J.; Yang, Y.; Dai, A.; Doshi, R.; Huang, A.; Song, Y.; Gehlhar, R.; Ames, A. D.; Gao, W., Biofuel-powered soft electronic skin with multiplexed and wireless sensing for human-machine interfaces. *Science Robotics* **2020,** *5* (41), eaaz7946. DOI: 10.1126/scirobotics.aaz7946.
35. Jeon, C.; Koo, J.; Lee, K.; Lee, M.; Kim, S. K.; Shin, S.; Hahn, S. K.; Sim, J. Y., A Smart Contact Lens Controller IC Supporting Dual-Mode Telemetry With Wireless-Powered Backscattering LSK and EM-Radiated RF Transmission Using a Single-Loop Antenna. *IEEE Journal of Solid-State Circuits* **2020,** *55* (4), 856-867.
36. Dautta, M.; Alshetaiwi, M.; Escobar, J.; Tseng, P., Passive and wireless, implantable glucose sensing with phenylboronic acid hydrogel-interlayer RF resonators. *Biosensors and Bioelectronics* **2020,** *151*, 112004-112011.
37. Cheng, X.; Wang, B.; Zhao, Y.; Hojaiji, H.; Lin, S.; Shih, R.; Lin, H.; Tamayosa, S.; Ham, B.; Stout, P.; Salahi, K.; Wang, Z.; Zhao, C.; Tan, J.; Emaminejad, S., A Mediator-Free Electroenzymatic Sensing Methodology to Mitigate Ionic and Electroactive Interferents' Effects for Reliable Wearable Metabolite and Nutrient Monitoring. *Advanced Functional Materials* **2020,** *30* (10), 1908507-1908518.

38. Yu, Y.; Nguyen, T.; Tathireddy, P.; Roundy, S.; Young, D. J., An <italic>In-Vitro</italic> Study of Wireless Inductive Sensing and Robust Packaging for Future Implantable Hydrogel-Based Glucose Monitoring Applications. *IEEE Sensors Journal* **2020,** *20* (4), 2145-2155.
39. Hassan, R. S.; Lee, J.; Kim, S., A Minimally Invasive Implantable Sensor for Continuous Wireless Glucose Monitoring Based on a Passive Resonator. *IEEE Antennas and Wireless Propagation Letters* **2020,** *19* (1), 124-128.
40. Mujeeb-U-Rahman, M.; Nazari, M. H.; Sencan, M.; Antwerp, W. V., A Novel Needle-Injectable Millimeter scale Wireless Electrochemical Glucose Sensing Platform for Artificial Pancreas Applications. *Scientific Reports* **2019,** *9* (1), 17421-17431.
41. García-Carmona, L.; Martín, A.; Sempionatto, J. R.; Moreto, J. R.; González, M. C.; Wang, J.; Escarpa, A., Pacifier Biosensor: Toward Noninvasive Saliva Biomarker Monitoring. *Analytical Chemistry* **2019,** *91* (21), 13883-13891.
42. Ma, J.; Jiang, Y.; Shen, L.; Ma, H.; Sun, T.; Lv, F.; Kiran, A.; Zhu, N., Wearable biomolecule smartsensors based on one-step fabricated berlin green printed arrays. *Biosensors and Bioelectronics* **2019,** *144*, 111637-111643.
43. Kim, S.-K.; Jeon, C.; Lee, G.-H.; Koo, J.; Cho, S. H.; Han, S.; Shin, M.-H.; Sim, J.-Y.; Hahn, S. K., Hyaluronate–Gold Nanoparticle/Glucose Oxidase Complex for Highly Sensitive Wireless Noninvasive Glucose Sensors. *ACS Applied Materials & Interfaces* **2019,** *11* (40), 37347-37356.
44. Zahertar, S.; Wang, Y.; Tao, R.; Xie, J.; Fu, Y. Q.; Torun, H., A fully integrated biosensing platform combining acoustofluidics and electromagnetic metamaterials. *Journal of Physics D: Applied Physics* **2019,** *52* (48), 485004. DOI: 10.1088/1361-6463/ab3f7d.
45. Sempionatto, J. R.; Brazaca, L. C.; García-Carmona, L.; Bolat, G.; Campbell, A. S.; Martin, A.; Tang, G.; Shah, R.; Mishra, R. K.; Kim, J.; Zucolotto, V.; Escarpa, A.; Wang, J., Eyeglasses-based tear biosensing system: Non-invasive detection of alcohol, vitamins and glucose. *Biosensors and Bioelectronics* **2019,** *137*, 161-170.
46. Mercer, C.; Bennett, R.; Conghaile, P. Ó.; Rusling, J. F.; Leech, D., Glucose biosensor based on open-source wireless microfluidic potentiostat. *Sensors and Actuators B: Chemical* **2019,** *290*, 616-624.
47. Xu, G.; Cheng, C.; Liu, Z.; Yuan, W.; Wu, X.; Lu, Y.; Low, S. S.; Liu, J.; Zhu, L.; Ji, D.; Li, S.; Chen, Z.; Wang, L.; Yang, Q.; Cui, Z.; Liu, Q., Battery-Free and Wireless Epidermal Electrochemical System with All-Printed Stretchable Electrode Array for Multiplexed In Situ Sweat Analysis. *Advanced Materials Technologies* **2019,** *4* (7), 1800658-1800670.
48. Lu, Y.; Jiang, K.; Chen, D.; Shen, G., Wearable sweat monitoring system with integrated micro-supercapacitors. *Nano Energy* **2019,** *58*, 624-632.
49. Toi, P. T.; Trung, T. Q.; Dang, T. M. L.; Bae, C. W.; Lee, N.-E., Highly Electrocatalytic, Durable, and Stretchable Nanohybrid Fiber for On-Body Sweat Glucose Detection. *ACS Applied Materials & Interfaces* **2019,** *11* (11), 10707-10717.
50. Liang, Y.; Ma, M.; Zhang, F.; Liu, F.; Liu, Z.; Wang, D.; Li, Y., An LC Wireless Microfluidic Sensor Based on Low Temperature Co-Fired Ceramic (LTCC) Technology. *Sensors* **2019,** *19* (5), 1189-1199.
51. Kim, K. B.; Lee, W.-C.; Cho, C.-H.; Park, D.-S.; Cho, S. J.; Shim, Y.-B., Continuous glucose monitoring using a microneedle array sensor coupled with a wireless signal transmitter. *Sensors and Actuators B: Chemical* **2019,** *281*, 14-21.
